# Improved Analysis of Isomeric Polyphenol Dimers Using the 4th Dimension of Trapped Ion Mobility Spectrometry—Mass Spectrometry

**DOI:** 10.3390/molecules27134176

**Published:** 2022-06-29

**Authors:** Aécio L. de Sousa Dias, Arnaud Verbaere, Emmanuelle Meudec, Stacy Deshaies, Cédric Saucier, Véronique Cheynier, Nicolas Sommerer

**Affiliations:** 1 SPO Sciences Pour l’Œnologie, Université de Montpellier, INRAE, Institut Agro, F-34060 Montpellier, France; aecio-luis.de-sousa-dias@inrae.fr (A.L.d.S.D.); arnaud.verbaere@inrae.fr (A.V.); emmanuelle.meudec@inrae.fr (E.M.); deshaiesstacy@gmail.com (S.D.); cedric.saucier@umontpellier.fr (C.S.); veronique.cheynier@inrae.fr (V.C.); 2 INRAE, PROBE Research Infrastructure, PFP Polyphenol Analysis Facility, F-34060 Montpellier, France

**Keywords:** ion mobility spectrometry, dehydrodicatechins, flavan-3-ol oxidation products, grape seed extract

## Abstract

Dehydrodicatechins resulting from (epi)catechin oxidation have been investigated in different foods and natural products, but they still offer some analytical challenges. The purpose of this research is to develop a method using ultra-high performance liquid chromatography coupled with trapped ion mobility spectrometry and tandem mass spectrometry (UHPLC−ESI−TIMS−QTOF−MS/MS) to improve the characterization of dehydrodicatechins from model solutions (oxidation dimers of (+)-catechin and/or (−)-epicatechin). Approximately 30 dehydrodicatechins were detected in the model solutions, including dehydrodicatechins B with *β* and *ε*-interflavanic configurations and dehydrodicatechins A with *γ*-configuration. A total of 11 dehydrodicatechins B, based on (−)-epicatechin, (+)-catechin, or both, were tentatively identified in a grape seed extract. All of them were of *β*-configuration, except for one compound that was of *ε*-configuration. TIMS allowed the mobility separation of chromatographically coeluted isomers including dehydrodicatechins and procyanidins with similar MS/MS fragmentation patterns that would hardly be distinguished by LC-MS/MS alone, which demonstrates the superiority of TIMS added to LC-MS/MS for these kinds of compounds. To the best of our knowledge, this is the first time that ion mobility spectrometry (IMS) was applied to the analysis of dehydrodicatechins. This method can be adapted for other natural products.

## 1. Introduction

Polyphenols are a large group of plant secondary metabolites [1]. Among them, flavan-3-ols are particularly abundant in foods and natural products. They are mostly present as oligomers and polymers, called proanthocyanidins whose degree of polymerization can be higher than 30 [2]. Flavan-3-ols and especially proanthocyanidins, have a sensory influence on food products contributing to bitterness and astringency [3] and indirectly to color [4,5]. They also have health benefits such as anti-cancer activities mainly related to their antioxidant and anti-inflammatory properties [6]. Flavan-3-ols can undergo autoxidation [7] and enzymatic oxidation by polyphenoloxidase (PPO), laccase, tyrosinase, and peroxidase. In particular, oxidation of (+)-catechin [8,9,10,11] and (−)-epicatechin [4] were reported to form dehydrodicatechins and other dehydrocatechin oligomers. These compounds are also associated with the sensory qualities and can be investigated as oxidation markers in food and natural products [11].

Structurally, procyanidin dimers are dimers of (+)-catechin and/or (−)-epicatechin formed by an interflavanic linkage (IFL) between the C4 position of a flavan-3-ol unit and the A-ring of the other unit in the position C6 or C8, forming a compound called B-type procyanidin with an exact mass of 578.1424 u. They can also have an additional C-O-C IFL forming an A-type procyanidin that has an exact mass of 576.1268 u because of its superior oxidation state. Dehydrodicatechins differ from procyanidins as they show a biphenyl or biphenyl ether IFL between the B-ring and the C6 or C8 positions of the A-ring (dehydrodicatechin B) [9] eventually with an additional C-O-C IFL and further rearrangement (dehydrodicatechin A) [12,13]. Dehydrodicatechins B and A are isomers of B-type and A-type procyanidins, respectively.

**Figure 1:**
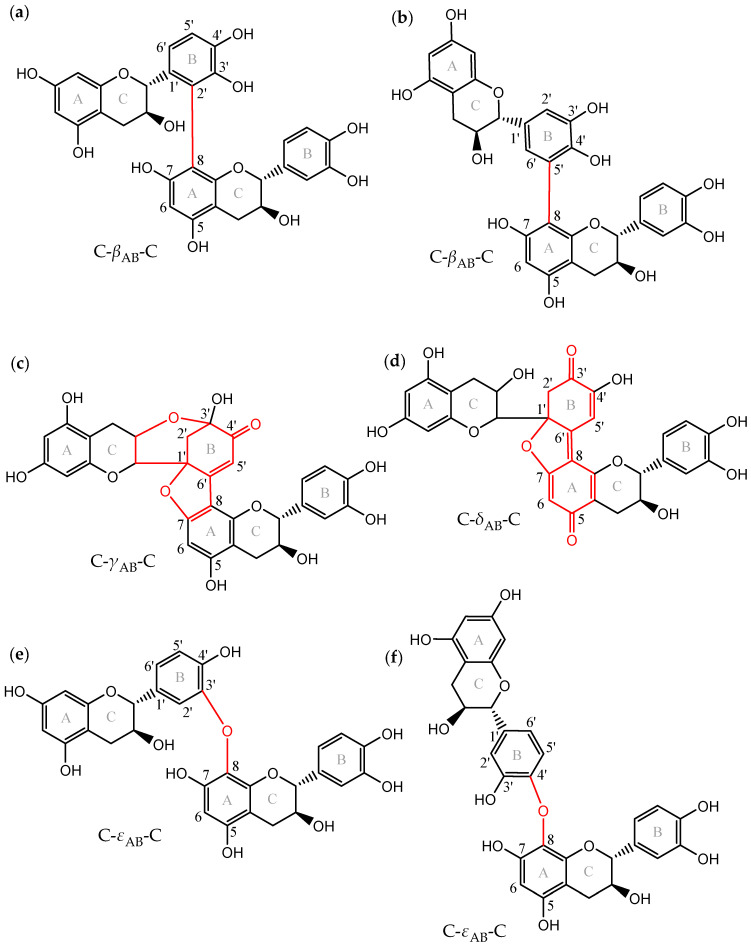
Dehydrodicatechins with different interflavanic configurations (in red): (**a**,**b**) compounds with *β*-configuration, (**c**) *γ*-configuration, (**d**) *δ*-configuration, and (**e**,**f**) *ε*-configuration. Exact mass is 578.1424 u for (**a**,**b**,**e**,**f**) and 576.1268 u for (**c**,**d**), corresponding to superior oxidation states. Nomenclature is briefly described in the Introduction section and proposed by Verloop and co-workers [14]. (**a**–**c**,**e**,**f**) correspond to N2, N4, N8, N3, and N6 standards, respectively, that were used in the present work. Adapted from Deshaies and co-workers [15] and Verloop and co-workers [14].

Another dehydrodicatechin nomenclature was proposed by Verloop and co-workers [14] based on the interflavanic configuration types and it was used in the present work. Figure 1 shows the interflavanic configurations that are energetically most favorable taking as an example structures of dehydrodicatechins based on (+)-catechin units. This includes compounds with *β*-configuration consisting of a biphenyl IFL (Figure 1a,b); *γ*-configuration with a biphenyl ether and a biphenyl IFLs, plus an additional intraflavanic ether linkage from the C-ring to the B-ring and a carbonyl group for one of the catechin units, resulting from intramolecular rearrangement (Figure 1c); and *δ*-configuration with an biphenyl ether and a double C-C bond in the IFL, plus a carbonyl group for both of the units resulting from intramolecular rearrangement (Figure 1d); and *ε*-configuration having a biphenyl ether IFL (Figure 1e,f). The *α*-configuration consists in a biphenyl and a biphenyl ether IFL that is not represented in Figure 1 because it is an unstable intermediate product formed after oxidation of a *β*-configuration dehydrodicatechin and quickly rearranges to form dehydrodicatechin of *γ-* or *δ*-configuration [10]. Compounds of *β-*, *γ-*, *δ-,* and *ε*-configuration of Figure 1 are named as C-*β*_AB_-C (Figure 1a,b), C-*γ*_AB_-C (Figure 1c), C-*δ*_AB_-C (Figure 1d), and C-*ε*_AB_-C (Figure 1e,f) according to Verloop and co-workers [14]. Where C (or EC) is the (+)-catechin (or (−)-epicatechin) unit, Greek letters correspond to the interflavanic configurations, subscript A and B letters correspond to A- and B-rings of the terminal and extension units, respectively. Terminal unit refers to the unit with the unreacted B-ring. The other unit of the compound is the extension unit.

Dehydrodicatechins have been analyzed by LC-MS/MS in different matrices such as black tea [14], green tea [4], beer [16], processed cocoa powders [17], and in wines and grape seed extracts [15]. This last work identified one dehydrodicatechin B and two dehydrodicatechins A, all derived from (+)-catechin and at low concentrations. The main analytical challenges for these compounds are related to the presence of numerous isomers [14], often found along with their procyanidin isomers in different matrices, such as in grape seed extracts [18], and are usually present in low concentrations and reference compounds are not easily available.

To improve characterization, Deshaies and co-workers [11] isolated eight fractions of (+)-catechin-derived dehydrodicatechins (six fractions containing dehydrodicatechins B and two corresponding to dehydrodicatechins A) from which five of them (N2, N3, N4, N6, and N8 standards) were fully characterized [11] (Figure 1). These fractions were used in the present work as reference compounds, and in another work using an UHPLC-Q-Orbitrap where three dehydrodicatechins were identified corresponding to N6, N7, and N8 standards. These fractions were produced from (+)-catechin and a grape PPO extract or laccase from *Trametes versicolor* at pH 3.6 using an incubation of 2 h and at room temperature [11]. Guyot and co-workers [10] also isolated six dehydrodicatechin B fractions and two dehydrodicatechin A fractions, produced at pH 3 and six using a grape PPO extract and an incubation of 1 h at room temperature. These oxidation systems are also useful to produce reference compounds in order to develop analytical methods. Additionally, Sun and co-workers reported that the negative ionization mode had higher selectivity and sensitivity than the positive ion mode [19]. Furthermore, specific MS/MS fragments were proposed to distinguish the different dehydrodicatechin interflavanic configurations [14] and to distinguish dehydrodicatechins from procyanidin dimers [13,14,19]. However, it is not possible to distinguish these compounds when they are chromatographically coeluted and have similar MS/MS fragmentation patterns using LC-MS/MS.

Ion mobility spectrometry (IMS) separates ions in the gas phase according to their charge, size, and shape, in combination with mass spectrometry (MS) and can potentially improve dehydrodicatechin analysis. This technique has been used to separate isomers and isobars, ameliorate compound annotation using the collision cross section as an additional identification criterium [20] and enhance the signal-to-noise ratio in the MS spectrum [21]. Trapped ion mobility spectrometry (TIMS), that separates ions by holding them using an electric field against a moving gas [22], has shown in many cases a greater resolving power compared with other IMS approaches [23]. IMS has been applied to polyphenols of different matrices such as herbal liqueurs, berries, and several natural products [20,24,25,26] including the separation of black tea isomeric theasinensins which are oxidation products from pyrogallol-type flavan-3-ols, with structures similar to dehydrodicatechins [27] and to grape seed extract procyanidins [28]. However, to the best of our knowledge, no study has been published on the application of IMS to dehydrodicatechins. This work aims to develop an UHPLC−ESI−TIMS−QTOF−MS/MS method for the analysis of dehydrodicatechin obtained by oxidation of (+)-catechin, (−)-epicatechin, or both of them using grape PPO in model systems and applying it to a grape seed extract.

## 2. Results and Discussion

### 2.1. Development of the UHPLC−ESI−TIMS−QTOF−MS Method

N1-N8 reference compounds, samples obtained by oxidation of the flavan-3-ols ((+)-catechin, (−)-epicatechin, or a (+)-catechin/(−)-epicatechin mixture) in model systems, and flavan-3-ols standards ((+)-catechin and (−)-epicatechin), procyanidins B1–B5) were individually analyzed by UHPLC−ESI−TIMS−QTOF−MS.

Both positive and negative ionization modes were tested. However, only the negative mode was selected because it showed better sensitivity and selectivity as shown earlier [19] and promoted the formation of specific MS/MS fragments of B-type dehydrodicatechins as described in previous works [14,19]. These specific fragments are discussed in the next section.

The collision energy was optimized to 27.3 eV. Under these conditions, the residual abundance of precursor ion was 20% for procyanidins, 100% for dehydrodicatechins of *β-* and *γ*-configuration and 10% for dehydrodicatechin of *ε*-configuration (Figure S1).

In terms of TIMS data acquisitions, the ion mobility data were first obtained from a survey mode using wide ranges of mobility, typically 0.3 to 1.9 inverse reduced mobility (1/K_0_) and 49 milliseconds (ms) ramp time. These parameters were tested on a mixture produced from different solutions described in the Experimental section. The three samples of the flavan-3-ol oxidation models were mixed in equal proportion. In this mixture, aliquots of solutions of procyanidins B1 and B3 were added so that their chromatographic peak areas were in the same order as those of other peaks of the mixture. Only these procyanidins were used because they were partly coeluted in preliminary tests. We used an approach based on a heat map graph to visualize and optimize the separations. This graph shows the mobility values in the y-axis and retention time (Rt) values in the x-axis for the ions of interest. These ions are selected to be shown on the graph by specifying a range of masses during data handling. For example, Figure S2 shows heat maps for the mass ranges of *m*/*z* 573.1–577.2, 861.1–865.3, and 1147.1–1153.3. Accumulation of detected ions of nearby mobilities and Rts in the heat map form regions called spots. In this Figure, the mass range of *m*/*z* 573.1–577.2 includes the *m*/*z* values of dimeric compounds such as dehydrodicatechins of *β-*, *γ-*, *δ-,* and *ε*-configurations and procyanidins B1 and B3. Moreover, the additional compounds detected in the mass ranges of *m*/*z* 861.1–865.3 and 1147.1–1153.3 correspond to dehydrotricatechins and dehydrotetracatechins, respectively, that were also formed in the oxidation model systems [14]. As observed in Figure S2, the spots corresponding to dimeric compounds had mainly mobility values between 1.0 and 1.25 1/K_0_, whereas the compounds having masses corresponding to dehydrotricatechins and dehydrotetracatechins showed higher mobility values. Procyanidins B1, B3, and one dehydrodicatechin were slightly separated by TIMS, whereas they were coeluted by chromatography. To improve this separation, smaller mobility intervals (up to 1.0−1.25 1/K_0_) and larger ramp time values (up to 835 ms) were tested. A better separation for dimeric compounds was obtained for values around 1.0−1.25 1/K_0_ and 512 ms, but there was a loss of sensitivity. Accordingly, ramp time was decreased to improve the sensitivity. The final parameters were 1.0−1.25 1/K_0_ and 150 ms for which a good general ion mobility separation and sensitivity were obtained.

Additionally, in terms of chromatographic analysis, the relative errors of Rt were calculated from the following formula: (Rt replicated 1 − Rt replicated 2)/Rt replicated 1 × 100). The relative errors of Rt were less than 0.36%. The same was observed for all other analysis performed in this work. The separated and tentatively identified compounds in the flavan-3-ols model solutions and in the grape seed extract are discussed in the next sections.

### 2.2. Analysis of Dehydrodicatechin Reference Compounds

Samples of reference compounds that were described in Experimental section were individually analyzed by UHPLC−ESI−TIMS−QTOF−MS using the optimized parameters. Figure S3 illustrates the approach used in this work for the reference compound N2, a dehydrodicatechin B. Extracted ion chromatograms (EIC) from the mass range of *m*/*z* 577.1–577.2 for the observation of dehydrodicatechins B (or *m*/*z* 575.1–575.2 for dehydrodicatechin A) were obtained. The compound or compounds of each chromatographic peak were also analyzed by TIMS and were detected in the form of spots in the heat map that was filtered to show only *m*/*z* values within the mass range of *m*/*z* 577.1–577.2 (or 575.1–575.2). From each spot, MS and MS/MS spectra were acquired from high resolution mass spectrometry (HRMS). Table S1 shows the Rt values of the chromatographic peaks as well as the mobility, MS, and MS/MS data of the spots for the reference compounds.

LC-MS/MS data from the most intense spots were first evaluated in order to determine specific fragments because they generally had more fragments in their MS/MS spectra. The peak of the N2 standard (Rt = 5.8 min) showed two spots at 1.099 and 1.185 1/K_0_ (Table S1). As expected, the MS spectrum of the spot at 1.1099 1/K_0_ showed the monocharged ion at *m*/*z* 577.1343 (100%) corresponding to the formula ([C_30_H_25_O_12_]^−^, with error of 1.5 ppm) of a dehydrodicatechin B (Table S1). This ion produced the main fragments at *m*/*z* 439.1026 [M − H − 138]^−^, *m*/*z* 425.0875 [M − H − 152]^−^ (retro-Diels–Alder (RDA) fragments), but also at *m*/*z* 393.0973 [M − H − 184]^−^, *m*/*z* 559.1245 [M − H − H_2_O]^−^ and *m*/*z* 533.1436 [M − H − CO_2_]^−^ (Table S1). These fragments are in agreement with the *β*-configuration [19] of this compound with a C2′(B-ring)−C8(A-ring) IFL. Figure S4 shows a scheme of the MS/MS fragmentation pathway for the N2 standard. The fragment ion at *m*/*z* 393.0973 was reported to be produced by benzopyran removal [19] having the [C_21_H_13_O_8_]^−^ formula. However, in the present work the HRMS data suggested the [C_22_H_17_O_7_]^−^ (1.8 ppm) formula which also corresponds to a C-ring fragmentation in the lower (+)-catechin unit followed by a water loss [4]. The spot with the highest mobility value at 1.185 1/K_0_ showed an ion at *m*/*z* 577.1341 (100%), but no MS/MS spectrum can be obtained from this ion probably due to its low concentration. Moreover, no in-source fragments were observed in the MS spectrum. As the N2 compound was obtained as a pure fraction [11] and the two spots of the N2 peak have the same Rt, they can correspond to protomers.

The peak of the N3 standard (Rt = 6.7 min) had two spots on the heat map (Table S1). The most intense spot at 1.076 1/K_0_ showed an ion at *m*/*z* 577.1343 (100 %) in its MS spectrum. In addition, the MS/MS spectrum revealed the presence of abundant fragments at *m*/*z* 289.0719 (44%) and *m*/*z* 288.0637 (100%) (Table S1). Both of the fragments correspond to a (+)-catechin unit, the last one being a radical anion and they indicate an *ε*-configuration MS/MS signature, as expected from the structure of the N3 compound (Figure S5) [10,14]. An additional fragment ion at *m*/*z* 425.0869 corresponded to an RDA fragment also observed in the N2 compound (Figure S5). Similarly, MS/MS data from the spot at 1.062 1/K_0_ corresponded to an *ε*-configuration (Table S1). Nevertheless, these data did not show the above-mentioned radical anion, probably because of the instability of this species or lower intensity of this spot. As for the N2 compound, these two spots of the N3 compound can correspond to protomers. In the same way, the single spot from the peak of the N6 compound also had HRMS data and MS/MS fragmentation pattern consistent with a dehydrodicatechin of *ε*-configuration, but additionally another fragment ion at *m*/*z* 439.1037 was detected, corresponding to an RDA fragment (Table S1 and Figure S7). These dehydrodicatechins of *ε*-configuration formed by (+)-catechin units exhibited higher Rts in relation to the Rt of the N2 standard, as reported earlier [11,15]. Finally, the N3 and N6 compound showed similar mobility values indicating that they have similar molecular shapes.

The peak related to the N4 standard (Rt = 9.2 min) consists in a mixture of isomers having a *β*-configuration of which one was fully characterized as shown in Figure 1 [11]. This peak showed one highly intense spot at 1.084 1/K_0_ that showed an MS spectrum containing an ion at *m*/*z* 577.1341 (100%) (Table S1). The MS/MS spectrum of this ion is in agreement with a *β*-interflavanic configuration (Figure S6). The spots at 1.102, and 1.122 1/K_0_ from this peak also had similar MS/MS spectra, whereas the one at 1.155 1/K_0_ showed only the RDA fragments at *m*/*z* 425.0844 and 439.1032 (Table S1) and corresponded to the less intense spot. The absence of the ion at *m*/*z* 289 or the radical anion at *m*/*z* 288 for this last spot is also in agreement with a compound of the *β*-configuration. Nonetheless, it was not possible to determine which of these other minor spots corresponded to protomers or to the isomer of the mixture from the MS data. The types of major adduct ions in the four spots are different. The spot at 1.084 1/K_0_ had no adduct ions, whereas the spots at 1.102 and 1.122 1/K_0_ had the additional ions at *m*/*z* 1155.2754 (14%) and *m*/*z* 1155.2755 (13%), respectively, corresponding to [2M − H]^−^. The spot at 1.155 1/K_0_ equally had an ion at *m*/*z* 1155.2745 (14%) [2M − H]^−^, but also the additional adduct ion at *m*/*z* 667.1305 (9). This ion is probably an adduct whose the most probable molecular formula proposed from the HRMS data were [C_32_H_27_O_16_]^−^ (−0.0 ppm). This adduct ion may be formed from the oxidized form [(M − 2H) − H]^−^ that is typically produced from *ortho*-diphenols [29] in combination with two formic acid molecules resulting in the species [(M − 2H) + 2HCOOH − H]^−^ (Table S1).

Likewise, the N5 compound peak (Rt = 10.2 min) and its single spot (Table S1) showed an ion at *m*/*z* 577.1352 (100%) whose MS/MS fragmentation pattern also corresponded to a *β*-configuration as expected for this compound (Table S1).

The N1 fraction corresponded to three close peaks that were called N1a, N1b, and N1c peaks (Table S1). The N1a peak (Rt = 5.2 min) was separated into three spots in the heat map whose mobility values are provided in Table S1. The MS spectrum of the spot at 1.128 1/K_0_ showed the monocharged ion at *m*/*z* 577.1338 (100%) corresponding to the formula of a dehydrodicatechin B (Table S1). The MS/MS fragmentation pattern of this ion corresponded to a dehydrodicatechins of *β*-configuration [19]. The spot showing the lowest mobility value at 1.064 1/K_0_ had an ion at *m*/*z* 577.1337 (100%) in its MS spectrum whose MS/MS fragmentation pattern was similar to the previously mentioned, also indicating a *β*-configuration (Table S1). The spot with the highest mobility value at 1.191 1/K_0_ showed an ion at *m*/*z* 577.1340 (100%), but no MS/MS spectrum of quality was obtained from this ion, probably due to its low concentration. Moreover, no in-source fragments were observed in the MS spectrum. The [M − H]^−^ ions corresponding to the dehydrodicatechin of the three spots obtained by TIMS separation correspond to molecules with different shapes, the species with higher mobility values corresponding to a more unfolded structure. The three spots showed different adduct patterns in their MS spectra as observed in Figure S10. The spot at 1.064 1/K_0_ had high abundance of an adduct ion at *m*/*z* 1155.2740 (54%) [2M − H]^−^ whereas this adduct was almost absent in the MS spectra obtained from the other two spots. On the other hand, the spot at 1.191 1/K_0_ additionally showed an abundant ion at *m*/*z* 667.1291 (32%) [(M − 2H) + 2HCOOH − H]^−^. This information on major adducts from different spots can be useful as a characteristic of the N1a peak in a sample. However, the spots of greater and lower mobility may not be detected in a less concentrated sample. In addition, the N1a peak showed a Rt close to procyanidin B3 (Rt = 5.3 min), as already reported for a dehydrodicatechin B [10,15]. Guyot and co-workers suggested that their dehydrodicatechin fraction coeluted with procyanidin B3 corresponded to a mixture of dehydrodicatechin of *β*-configuration based on (+)-catechin from the NMR, MS, and MS/MS spectra. Therefore, the ions [M − H]^−^ corresponding to dehydrodicatechins of each of the three spots of peak N1a may correspond to isomers and for two of them that showed MS/MS fragmentation data, a *β*-interflavanic configuration can be proposed. Another possibility is that these ions may correspond to protomers defined as isomers differing in the binding or removal site of a proton in the positive or negative ion mode, respectively [30,31].

N1b peak (Rt = 5.3 min) showed five spots (Table S1). The spots at 1.069, 1.110, 1.136 (the most intense spot), and 1.155 1/K_0_ showed the ion corresponding to the dehydrodicatechin formula whose *m*/*z* values ranged from *m*/*z* 577.1338 to *m*/*z* 577.1343 (100%) in their MS spectra. MS/MS fragmentation patterns of these ions were similar to those of the N1a peak, corresponding to the *β*-interflavanic configuration (Table S1). However, it was not possible to obtain a quality MS/MS spectrum for the ion at *m*/*z* 577.1342 (69%) of the spot at 1.187 1/K_0_ and no in-source fragmentation was observed in its MS spectrum. As for the N1a peak, the spots having higher and lower mobility showed different adducts in their MS spectra. The spot at 1.069 1/K_0_ showed the adduct ion at *m*/*z* 1155.2753 (9%) [2M − H]^−^ (Table S1) whereas the spot at 1.110 1/K_0_ had the adduct at 667.1299 (6%) ([(M − 2H) + 2HCOOH − H]^−^) and an abundant ion at *m*/*z* 1155.2745 (70%) [2M − H]^−^. On the other hand, the spot at 1.187 1/K_0_ showed the adduct at 667.1291 (100%) ([(M − 2H) + 2HCOOH − H]^−^ as the base peak. Finally, as for the N1a peak, the ions [M − H]^−^ of these five spots of the N1b peak may correspond to isomers and additionally four of them that showed MS/MS fragmentation data can have a *β*-configuration. These five ions may also correspond to protomers.

The N1c peak (Rt = 5.4 min) had two spots in the heat map at 1.113 and 1.164 1/K_0_. HRMS and MS/MS data (Table S1) from these spots also corresponded to dehydrodicatechins of *β*-configuration. As for N1a and N1b, the lowest spot of N1c at 1.113 1/K_0_ showed an additional adduct ion in its MS spectrum at *m*/*z* 1155.2757 (13%) [2M − H]^−^, whereas in the other N1c spot this ion was not observed (Table S1). Moreover, the [M − H]^−^ ions of these spots may correspond to isomers or protomers as discussed for N1a and N1b peaks. Deshaies and co-workers [15] observed two peaks for the N1 fraction. They used a different column and chromatographic method which may explain why in the present work we found three peaks for this fraction.

Among the dehydrodicatechins B, the deprotonated ion [M − H]^−^ of the spot of the N5 compound as well as the [M − H]^−^ ion at 1.185 1/K_0_ of the N2 compound had high mobility values indicating that they have a more unfolded structure. In contrast, the [M − H]^−^ dehydrodicatechin ions of the N6, N4, and N3 compounds and of one spot at 1.099 1/K_0_ of the N2 compound had low mobility values indicating a more compact structure. The [M − H]^−^ dehydrodicatechin ions of the spots of the N1c peak exhibited intermediate mobility values. Lastly, the N1a and N1b peaks showed spots containing [M − H]^−^ dehydrodicatechin ions throughout the entire range of mobility (between 1 and 1.25 1/K_0_).

The N8 (Figure 1) and N7 compounds correspond to dehydrodicatechins A which showed high Rts (Rt = 12.2 and 13.3 min, respectively) as observed in other works [10,11,15]. The peak of the N8 compound (Rt = 13.3 min) corresponding to a dehydrodicatechin of *γ*-configuration (Figure 1) had two spots in the heat map at 1.097 and 1.164 1/K_0_ (Table S1). The MS spectrum of the more intense spot at 1.164 1/K_0_ had an ion at *m*/*z* 575.1197 (100%). C-ring MS/MS fragmentation of the lower (+)-catechin unit of this compound can result in the following fragments (Table S1 and Figure S8 for a partial MS/MS fragmentation pathway): *m*/*z* 394.0694 [17], *m*/*z* 423.0719 [10] already was reported for a *γ*-configuration, and *m*/*z* 407.0765. The fragment ion at *m*/*z* 437.0864 can correspond to a C-ring fragmentation of the lower (+)-catechin unit whereas the fragment at *m*/*z* 449.0877 was likely formed from a loss of B-ring of the lower (+)-catechin unit, followed by a loss of H_2_O [14] or from a C-ring fragmentation in the upper (+)-catechin unit in combination with water loss [4] as were also found for dehydrodicatechins of *δ*-configuration. The less intense spot at 1.097 1/K_0_ showed only a fragment ion at *m*/*z* 449 probably due to the low intensity of the precursor ion. Accordingly, the MS/MS fragmentation patterns with the five fragments found for the spot at 1.164 1/K_0_ were used as a *γ*-configuration MS/MS signature. The [M − H]^−^ dehydrodicatechin ions of the two spots of the N8 compound are most likely protomers, as for the N2 and N3 compounds. It is worth mentioning that this MS/MS fragmentation pattern was quite different from one described for a dehydrodicatechin of *γ*-configuration, showing major fragment ions at *m*/*z* 411, *m*/*z* 492, *m*/*z* 287, and *m*/*z* 515 [14], possibly due to the different instruments and experimental conditions. Finally, the spot at 1.097 1/K_0_ had an additional ion in its MS spectrum at 1151.2453 (15) [2M − H]^−^ that was not found in the other spot for this compound (Table S1).

The peak of the N7 compound and its corresponding spot showed an ion at *m*/*z* 575.1196 (100%) (Table S1). Guyot and co-workers [10] proposed a *δ*-interflavanic configuration for the compound having the lower Rt. MS/MS fragments of N7 showed the fragments corresponding to the loss of H_2_O and CO_2_ (*m*/*z* 557.1102 and *m*/*z* 531.1322, respectively) as well as the ions at *m*/*z* 437.0858 and *m*/*z* 449.0866 as were also found and discussed for the N8 compound. Consequently, it was not possible to determine an interflavanic configuration for the peak N7 compound. It may be of *γ-* or *δ*-configuration.

In terms of mobility values, the [M − H]^−^ dehydrodicatechin ion of the N7 compound spot had a mobility value similar to that of the [M − H]^−^ ion of the N8 compound spot at 1.097 1/K_0_. The [M − H]^−^ dehydrodicatechin ion of the other N8 compound spot showed a higher mobility value at 1.164 1/K_0_.

Regarding procyanidin standards, they are isomers of dehydrodicatechins B and showed low Rt, except procyanidin B5 which had high Rt which is expected for a C4−C6 IFL (Table S1). All these compounds provide the main MS/MS fragments at *m*/*z* 451.10, 425.08, 407.07, and 289.07 (Table S1) that were already discussed in previous work for the ESI negative mode [19]. An example of the MS spectrum is shown in Figure S2 and a partial MS/MS fragmentation pathway is shown in Figure S9 for procyanidin B3. The ion at *m*/*z* 451.10 was reported as specific for procyanidins in comparison with dehydrodicatechins B. Other fragments such as *m*/*z* 559.12 and 533.14 corresponding to H_2_O and CO_2_ losses were not always found. Moreover, a low intensity spot of procyanidin B1 at 1.128 1/K_0_ showed only the fragment ion at *m*/*z* 425.0878. This spot can be a protomer of the other spot of this compound. The procyanidins exhibited similar mobility values, except for procyanidin B5 which showed the highest value.

### 2.3. Oxidation Dimers of (+)-Catechin

**Figure 2:**
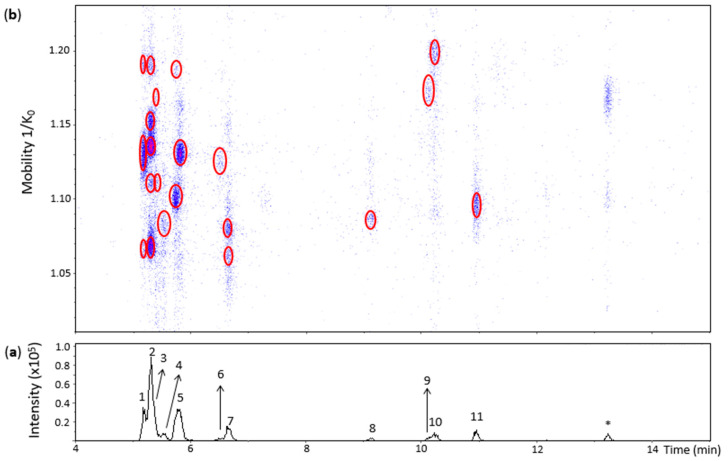
UHPLC−ESI (−)−TIMS−QTOF−MS/MS analysis of dehydrodicatechins in model solution containing (+)-catechin and grape PPO extract. (**a**) EIC from the mass range of *m*/*z* 577.1–577.2; (**b**) Heat map showing the mobility versus Rt for the mass range of *m*/*z* 577.1–577.2. Full details on peak numbers are available in Table S2. * Signal of an isotopic ion at *m*/*z* 577 of *m*/*z* 575 that is found in Figure 3 (peak 14). Ellipses on the heat map mark the main spots.

Oxidation of (+)-catechin by grape PPO extract in an unbuffered aqueous solution (pH ~ 5) at room temperature for 1 h produced several oxidation dimers. Figure 2 shows an EIC and a heat map obtained from an *m*/*z* 577.1–577.2 range where the *m*/*z* value of dehydrodicatechins B is included. Eleven peaks were observed when considering even minor and unresolved peaks. The theoretical number of dehydrodicatechins B is 10, considering that the two constitutive units are linked through A-ring (C6 or C8 position)/B-ring (5 positions) bonds [14], suggesting that some of the peaks in Figure 2 may correspond to compounds containing (+)-epicatechin units resulting from (+)-catechin epimerization. This epimerization may have occurred during the sample preparation process. He and co-workers [13] also observed the epimerization process in dehydrodicatechins from their autoxidation models. Epimerization is also often reported for dimeric procyanidins [32].

**Peak 1** (Rt = 5.2 min) was separated into three spots (Figure 2) whose HRMS and MS/MS spectra were similar to the spots found for the N1a peak provided in Table S1. However, the spot showing the lowest mobility value at 1.062 1/K_0_ showed only the RDA MS/MS fragments at *m*/*z* 439.1026 and *m*/*z* 425.0869 probably due to the low abundance of the precursor ion (Table S2). Therefore, peak 1 annotation was the same as for the N1a peak. The three spots may be protomers or isomers and the spots at 1.062 and 1.129 1/K_0_ have a *β*-interflavanic configuration (Table S2)

In the same way, the mobility values and HRMS and MS/MS data from the five spots of **peak 2** (Rt = 5.3 min) (Figure 2 and Table S2) corresponded to the N1b peak of Table S1. Therefore, these five spots may correspond to protomers or isomers and the first four spots are dehydrodicatechin of *β*-configuration.

The UHPLC−ESI−TIMS−QTOF−MS method of the present work was able to detect two spots in the heat map even at low intensities of the **peak 3** that is coeluted with peak 2 (Figure 2 and Table S2) which demonstrates the interest of combining TIMS with LC-MS/MS. They corresponded to the spots of the N1c peak (Table S1). However, the spot at 1.111 1/K_0_ showed only the fragment ion at *m*/*z* 439.1004 and the spot at 1.165 1/K_0_ did not show the MS/MS spectrum due to the low intensities of these spots. As discussed for the N1c peak in the previous section, these spots may correspond to isomers or protomers of the *β*-configuration.

**Peak 5** (Rt = 5.8 min) showed three spots slightly misaligned in relation to the Rt (Figure 2) suggesting the presence of at least two compounds, confirming the potential of the additional IMS dimension to improve chromatographic analysis. The spots at 1.098 and 1.188 1/K_0_ corresponded to the N2 standard including the MS/MS fragmentation pattern of the first spot. Therefore, they were annotated as for the N2 standard (Table S2). The spot at 1.130 1/K_0_ also showed the MS/MS spectrum consistent with *β*-configuration, but it was not found in the heat map of the N2 standard (Figure S3). This spot can be attributed to an (+)-catechin-derived dehydrodicatechin or to a dehydrodicatechin resulting from the epimerization of one or two (+)-catechin units. The different compounds of peak 5 are not distinguished on the basis of LC-MS/MS alone, which reinforces the complementarity between IMS and LC-MS/MS.

**Peak 7** (Rt = 6.7 min) and **peak 11** (Rt = 11 min) showed spots on the heat map (Figure 2) with HRMS and MS/MS data corresponding to the N3 and N6 standards, respectively (Table S2) and consisted of dehydrocatechin of *ε*-configuration.

**Peak 8** (Rt = 9.2 min) revealed a poor heat map profile with low intensities where only one spot at 1.087 1/K_0_ is well-visible (Figure 2) that also showed a poor MS/MS spectrum (Table S2), corresponding to a dehydrodicatechin of *β*-configuration as observed for the N4 reference fraction (Table S1). In the same way, **peak 10** (Rt = 10.2 min) and its spot (Figure 2) corresponded to the N5 standard having a *β*-configuration (Table S2).

Our method allowed the detection of spots in the heat map even at low intensities that corresponded to poor chromatographic signals, such as peaks 4, 6, and 9, which illustrates the analytical improvement provided by TIMS. They did not correspond to any of the reference compounds of dehydrodicatechins formed from (+)-catechin units. Additionally, **peak 4** did not generate an MS/MS spectrum. Consequently, the interflavanic configuration of the of the compound of the spot of peak 4 could not be determined. This compound may correspond to a dehydrodicatechin based on (+)-catechin units or it may contain at least one (+)-epicatechin unit resulting from epimerization (Table S2).

Similarly, the spot at 1.125 1/K_0_ of **peak 6** showed the HRMS and MS/MS spectrum consistent with a dehydrodicatechin of *β*-configuration (Table S2). The corresponding compound may also be based on (+)-catechins or on one or two (+)-epicatechins formed by epimerization.

The single spot at 1.173 1/K_0_ of **peak 9** exhibited an MS/MS spectrum corresponding to a dehydrodicatechin of *ε*-configuration (Table S2). It may also correspond to a dehydrodicatechin based on (+)-catechin units or it may be formed by epimerization of this compound.

**Figure 3:**
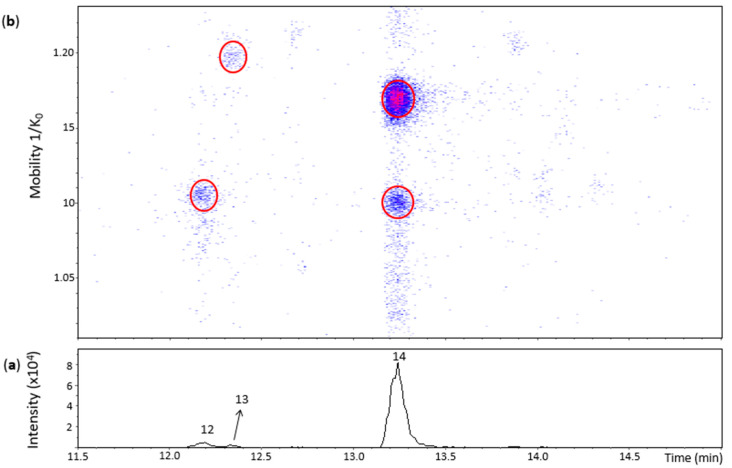
UHPLC−ESI (−)−TIMS−QTOF−MS/MS analysis of dehydrodicatechins in model solution containing (+)-catechin and grape PPO extract. (**a**) EIC from the mass range of *m*/*z* 575.1–575.2; (**b**) heat map showing the mobility versus Rt for the mass range of *m*/*z* 575.1–575.2. Full details on peak numbers are available in Table S2. Ellipses on the heat map mark the main spots.

Figure 3 shows three main chromatographic peaks and their corresponding spots in the heat map for an *m*/*z* 575.1–575.2 range. **Peak 14** (Rt = 13.2 min) had two spots in the heat map (Figure 3) corresponding to the N8 standard (Tables S1 and S2). The spot at 1.100 1/K_0_ showed a better MS/MS spectrum compared with the N8 standard, but without the fragment at *m*/*z* 423 found for the highest and more intense spot of the N8 standard. Both of these spots were thus annotated as protomers of dehydrodicatechins of *γ*-configuration.

**Peak 12** (Rt = 12.2 min) and its spot (Table S2) corresponded to the N7 standard (Table S1). Nevertheless, the MS/MS experiment performed on this spot generated a poor MS/MS spectrum due to low intensity of the spot and only the fragment ion at *m*/*z* 557.1070 ([C_30_H_21_O_11_]^−^, 3.4 ppm) (Table S2) can be proposed as a loss of water. Even without a good MS/MS spectrum, this spot may correspond to a dehydrodicatechin of *γ-* or *δ*-configuration, as discussed in the previous section for the N7 standard.

The small **peak 13** also had a low intensity spot at 1.197 1/K_0_ (Table S2). This spot had no MS/MS fragmentation spectrum and did not correspond to a reference compound. Consequently, the interflavanic configuration could not be determined, but its corresponding dehydrodicatechin is likely formed by (+)-catechin units. Guyot and co-workers [10] and Deshaies and co-workers [11] isolated two fractions containing dehydrodicatechins A derived from (+)-catechins. In the present work, three peaks (peak 12, 13 and 14) were detected. This difference can be explained by the different experimental conditions as described in the Introduction section.

### 2.4. Oxidation Dimers of (−)-Epicatechin

**Figure 4:**
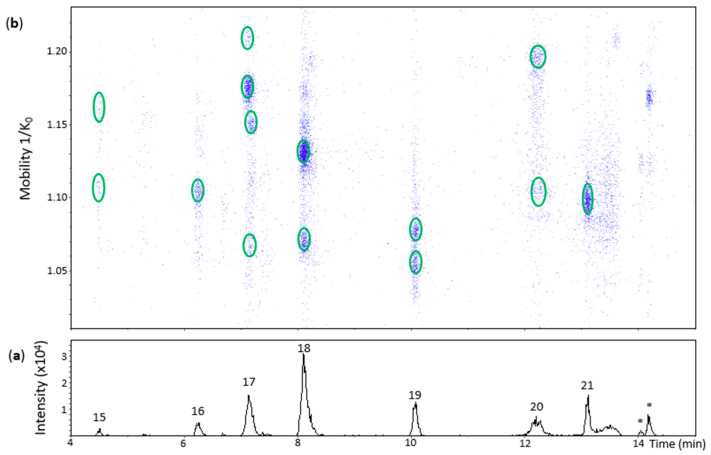
UHPLC−ESI (−)−TIMS−QTOF−MS/MS analysis of dehydrodicatechins in model solution containing (−)-epicatechin and grape PPO extract. (**a**) EIC from the mass range of *m*/*z* 577.1–577.2; (**b**) heat map showing the mobility versus Rt for the mass range of *m*/*z* 577.1–577.2. Full details on peak numbers are available in Table S2. * Signal of isotopic ion at *m*/*z* 577 of *m*/*z* 575 that is found in Figure 5 (peaks 23 and 24). Ellipses on the heat map mark the main spots.

**Figure 5:**
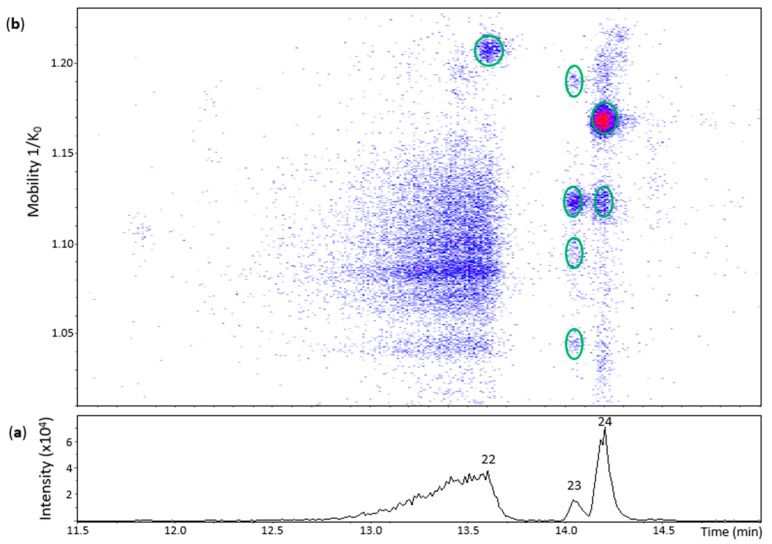
UHPLC−ESI (−)−TIMS−QTOF−MS/MS analysis of dehydrodicatechins in model solution containing (−)-epicatechin and grape PPO extract. (**a**) EIC from the mass range of *m*/*z* 575.1–575.2. (**b**) Heat map showing the mobility versus Rt for the mass range of *m*/*z* 575.1–575.2. Full details on peak numbers are available in Table S2. Ellipses on the heat map mark the main spots.

Oxidation of (−)-epicatechin produced 10 chromatographic peaks of different Rt (Figure 4 and Figure 5). **Peak 15** (Rt = 4.5 min) had two low intensity spots at 1.109 and 1.158 1/K_0_ that showed low quality MS/MS spectra with some fragments of a *β*-configuration dehydrodicatechin. The absence of the ions at *m*/*z* 289 and 288 and the low Rt values reinforces the hypothesis of *β*-configuration for these spots that may be protomers or isomers. Additionally, the spot at 1.109 1/K_0_ showed an MS spectrum with the adduct ion at *m*/*z* 645.1239 (7%) [(M − H) + Na + HCOO^−^]^−^.

**Peak 16** (Rt = 6.2 min), **peak 18** (Rt = 8.1 min) and **peak 20** (Rt = 12.2 min) showed one, two, and two spots, respectively (Figure 4). All of them had HRMS and MS/MS fragmentation patterns corresponding to dehydrodicatechins of *β*-configuration (Table S2). The additional spots are probably protomers or isomers with a same interflavanic configuration.

**Peak 17** (Rt = 7.2 min) corresponded to four spots on the heat map (Figure 4) slightly misaligned with respect to the Rt axis, suggesting the presence of more than one compound. The most intense spot at 1.175 1/K_0_ corresponded to a *β*-configuration compound (Table S2). The spot at 1.210 1/K_0_ had a different MS spectrum with highly abundant adduct ions at *m*/*z* 599.1165 (92%) [(M − H) – H + Na]^−^ and *m*/*z* 667.1312 (35%) [(M − 2H) + 2HCOOH − H]^−^ in addition to the ion at *m*/*z* 577.1357 (100%). It was not possible to obtain an MS/MS spectrum from this ion, probably due to the low abundance of MS signals. These two aligned spots may correspond to protomers or isomers. The spots at 1.073 1/K_0_ and 1.148 1/K_0_ showed ions at *m*/*z* 577.1342 (100%) and *m*/*z* 577.1347 (100%), respectively, along with poor MS/MS spectra for both of them with the fragments at *m*/*z* 425.08 and *m*/*z* 559.12 and absence of an *m*/*z* 289 fragment, suggesting they are also protomers or isomers of *β*-configuration.

**Peak 19** (Rt = 10.1 min) and **peak 21** (Rt = 13.1 min) corresponded to two spots and one spot, respectively (Figure 4), showing [M − H]^−^ ions ranging from at *m*/*z* 577.1350 to *m*/*z* 577.1356 (100%). MS/MS fragments from these spots (Table S2) indicate that they have *ε*-configuration. The dehydrodicatechins of peak 19 may be two protomers or isomers.

**Peak 22** (Rt = 13.6 min) had one spot in the heat map (Figure 5) whose ion at *m*/*z* 575.1200 (100%) corresponded to a dehydrodiepicatechin A (Table S2) and showed a poor MS/MS spectrum with no possibility to determine its configuration that may be of *γ-* or *δ*-configuration, as discussed for peak 12 of the (+)-catechin model.

**Peak 23** (Rt = 14.1 min) showed four spots (Figure 5) with ions ranging from *m*/*z* 575.1180 to *m*/*z* 575.1196 (100%) (Table S2). The spot with greater mobility value had an adduct ion at *m*/*z* 643.1068 (66%) [(M − H) + Na + HCOO^−^]^−^ ([C_31_H_24_NaO_14_]^−^, 0.3 ppm). In general, the first three spots corresponded to MS/MS fragments at *m*/*z* 437,08, *m*/*z* 449.08, and *m*/*z* 407.08 (but not all at the same time) (Table S2). Consequently, it was not possible to determine the interflavanic configuration, as discussed for peak 22, respectively, that may be of *γ-* or *δ*-configuration. It was not possible to obtain an MS/MS spectrum for the spot of higher mobility. The four spots may correspond to protomers or isomers. On the other hand, **peak 24** (Rt = 14.2 min) showed two spots at 1.119 and 1.168 1/K_0_ (Figure 5) with ions at *m*/*z* 575.1183 (100%) and *m*/*z* 575.1192 (100%), respectively, whose MS/MS spectrum corresponded to two compounds (protomers or isomers) of *γ*-configuration (Table S2).

In this oxidation model, 11 dehydrodicatechins were identified: 6, 2, and 1 of *β*, *ε,* and *γ*-configuration, respectively, without counting their respective protomers or isomers when that was the case. Slightly different results were obtained by Verloop and co-workers [14] who found five compounds of *β*-configuration and the same number of *ε*-configuration and another one of *δ*-configuration from an (−)-epicatechin oxidation model using a mushroom tyrosinase at pH 5.5 for 5 min at 25 °C. The different methodologies between the two works may explain the different results.

### 2.5. Oxidation Dimers of a Mixture of (+)-Catechin and (−)-Epicatechin

**Figure 6:**
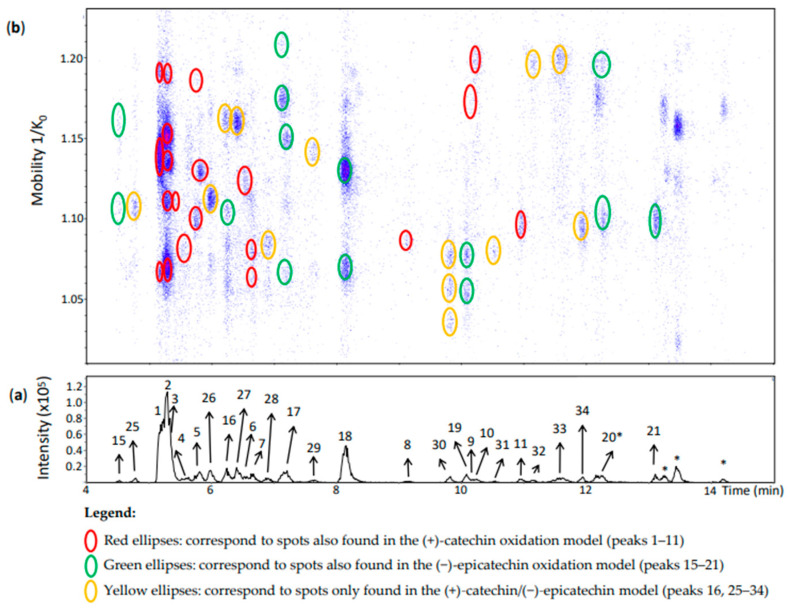
UHPLC−ESI (−)−TIMS−QTOF−MS/MS analysis of dehydrodicatechins in model solution containing (+)-catechin and (−)-epicatechin along with a grape PPO extract. (**a**) EIC from the mass range of *m*/*z* 577.1–577.2; (**b**) heat map showing the mobility versus Rt for the mass range of *m*/*z* 577.1–577.2. Full details on peak numbers are available in Table S2. * Signal of isotopic ion at *m*/*z* 577 of *m*/*z* 575 that is found in Figure 7 (peaks 14, 35 and 24). Ellipses on the heat map mark the main spots.

**Figure 7:**
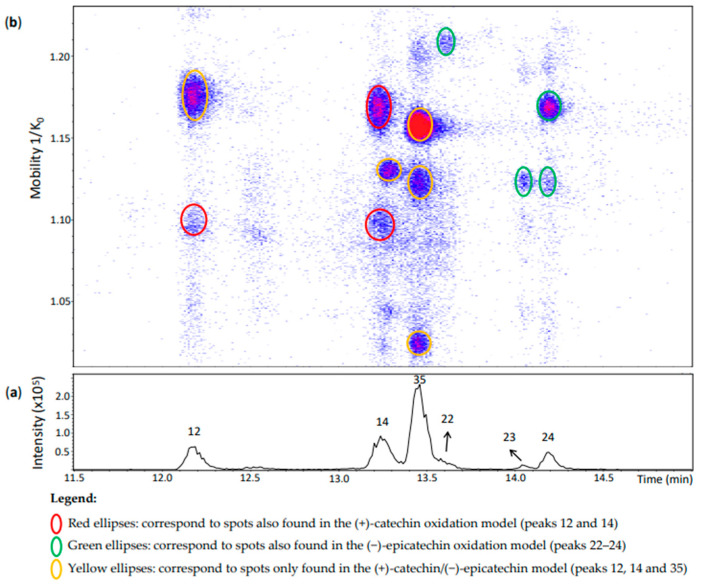
UHPLC−ESI (−)−TIMS−QTOF−MS/MS analysis of dehydrodicatechins in model solution containing (+)-catechin and (−)-epicatechin along with grape PPO extract. (**a**) EIC from the mass range of *m*/*z* 575.1–575.2. (**b**) Heat map showing the mobility versus Rt for the mass range of *m*/*z* 575.1–575.2. Full details on peak numbers are available in Table S2. Ellipses on the heat map mark the main spots.

The (+)-catechin/(−)-epicatechin model produced, as expected, additional chromatographic peaks (peaks 25–35) and spots in the heat map (Figure 6 and Figure 7) likely derived from both (+)-catechin and (−)-epicatechin.

**Peaks 25–29 and 31–33** (Figure 6) corresponded each to a single spot. According to the MS/MS fragmentation patterns previously discussed, these peaks were partly identified as *β*-type compounds from both flavan-3-ol monomers (Table S2). Despite the poor MS/MS spectrum of **peak 29**, it showed the *β*-configuration specific fragment at *m*/*z* 393. It is important to highlight that **peak 16** corresponded to two spots (Figure 6) which were also compatible with a *β*-configuration (Table S2), but one of them was an additional spot at 1.161 1/K_0_ compared with peak 16 of the (−)-epicatechin model (Figure 4). Therefore, it can be considered as derived from both (+)-catechin and (−)-epicatechin whereas the other spot can be referred to as an (−)-epicatechin dimer. In addition, **peaks 30 and 34** had three and one spot, respectively. All of them corresponded to an *ε*-configuration having (+)-catechin and (−)-epicatechin units. In the case of the three spots of peak 30 they may be protomers or isomers.

**Peak 1** corresponded to the same identification of peak 1 of the (+)-catechin model (Figure 6 and Table S2). Nevertheless, the possibility of this compound being coeluted with a dimer of (+)-catechin and (−)-epicatechin cannot be excluded. Indeed, Sun and co-workers [19] also identified two *β*-type dehydrodicatechins formed from two (+)-catechin units and from both (+)-catechin and (−)-epicatechin units having very close and low Rts and eluting before (+)-catechin. In this work, (+)-catechin and (−)-epicatechin eluted at 5.5 and 7.4 min, respectively.

Likewise, **peak 2** (Rt = 5.3 min) had five spots (Figure 6) with mobility values and HRMS and MS/MS spectra similar to those of peak 2 of the (+)-catechin oxidation model (Table S2). These five spots thus can contain the same compound as for peak 2 of the (+)-catechin model. As previously discussed for the peak 1, it is possible that these spots also contain a dehydrodicatechin of *β*-configuration formed from both (+)-catechin and (−)-epicatechin.

**Peak 3** (Rt = 5.4 min) showed one spot at 1.111 1/K_0_ that may correspond to one spot of the N1c peak found at 1.113 1/K_0_ (Tables S1 and S2). The MS/MS spectrum from the spot at 1.111 1/K_0_ corresponded to a *β*-configuration by the absence of the fragments at *m*/*z* 289 and 288 (Table S2). Therefore, peak 3 may be formed from (+)-catechin monomers and may have a *β*-configuration.

**Peak 4** corresponded to the peak 4 of the (+)-catechin model. Its interflavanic configuration could not be determined because of the absence of MS/MS spectrum. As for the (+)-catechin model, this peak may correspond to a dehydrodicatechin formed from (+)-catechin units or it may be the result of epimerization of this compound (Table S2).

**Peak 5, 10, and 11** were identified as the corresponding peaks of the (+)-catechin model (Table S2). Rt and mobility values for **peak 8** also corresponded to those of the (+)-catechin model. However, the MS/MS spectra were quite different. In the present model, there was an additional abundant fragment at *m*/*z* 289.0727 (42%) suggesting an *ε*-configuration. Therefore, peak 8 may correspond to a mixture of a dehydrodicatechin of *ε*-configuration formed from both (+)-catechin and (−)-epicatechin units with a dehydrodicatechin of *β*-configuration formed from (+)-catechins as also observed in the (+)-catechin model.

In the same way, **peaks 15, 17–19, and 21** were identified as the corresponding peaks of the (−)-epicatechin model (Table S2). The different spots of peak 15 and 17 showed a similar adduct profiles as observed for the (−)-epicatechin model. **Peak 20** showed two spots (Figure 6 and Table S2) as for the (−)-epicatechin model. The MS spectrum of the spot at 1.197 1/K_0_ showed not only the ion at *m*/*z* 577.1340 (100%) but also the ion at *m*/*z* 575.1178 (18%), corresponding to a dehydrodicatechin A. Indeed, this spot was partly coeluted with another one of a dehydrodicatechin A (Figure 7, peak 12). In addition, no MS/MS data were obtained for the ion at *m*/*z* 577.1340 from this spot probably because of the presence of an interfering ion at *m*/*z* 575 in the same mobility region that was preferentially fragmented. Consequently, the interflavanic configuration of the compound of this spot could not be determined. The spot at 1.097 1/K_0_ of peak 20 (Figure 6 and Table S2) showed an MS spectrum also containing an ion corresponding to a dehydrodicatechin A at *m*/*z* 575.1189 (100%) and an ion at *m*/*z* 577.1348 (50%). This last ion did not generate MS/MS data and no interflavanic configuration was proposed, as for the first discussed spot.

**Peaks 6 and 7** were annotated as for the (+)-catechin model based on their mobility, HRMS and MS/MS data (Figure 6 and Table S2); however, the interflavanic configuration of the dehydrodicatechin B of **peak 9** could not be determined because of the absence of MS/MS fragmentation from its spot that had low intensity.

**Peak 12** (Rt = 12.2 min) (Figure 7) showed a spot at 1.176 1/K_0_ that was not observed in the other models. It corresponded to an ion at *m*/*z* 575.1185 (100%) whose MS/MS fragmentation indicates a *γ*-interflavanic configuration (Table S2). On the other hand, the MS spectrum of another spot of this peak at 1.095 1/K_0_ had the ions at *m*/*z* 575.1190 (100%) whose isotopic profile contained an abundant ion at *m*/*z* 577.1335 (19%), suggesting that this last ion can be another compound. Indeed, it corresponds to the ion at *m*/*z* 577 previously discussed for peak 20 separated in the same region of mobility. MS/MS experiments on *m*/*z* 575 generated a different fragmentation pattern (Table S2). Fragments did not correspond to the reference compound N7 separated in the same mobility (Figure 2). This MS/MS fragmentation pattern did not correspond to any other reference compound used in this work that covered different interflavanic configurations. Additionally, the fragment at *m*/*z* 289 was not related to compounds of *δ-* and *γ*-configuration [14]. This ion at *m*/*z* 575.1190 may correspond to an unknown compound or to a mixture of compounds that were selected together during MS/MS experiments, which can explain its different MS/MS fragmentation pattern. In any case, this spot may include a dehydrodicatechin A.

**Peak 14** in Figure 7 showed three misaligned spots with respect to the Rt. The compound at 1.169 1/K_0_ corresponded to a *γ*-configuration product derived from two (+)-catechins as in the (+)-catechin model (Table S2). As mentioned above for peak 12, the MS/MS fragmentation pattern did not allow to determine the interflavanic configuration of the spot at 1.098 1/K_0_ which can also correspond to a mixture of compounds containing a dehydrodicatechin A. Finally, the compound at 1.129 1/K_0_ could be identified as a (+)-catechin/(−)-epicatechin derivative by comparing its mobility with those of the other models (Figure 3, Figure 5 and Figure 7 and Table S2). However, the interflavanic configuration of this compound could not be determined because of its poor MS/MS spectrum.

Similarly, the three main spots of **peak 35** correspond to (+)-catechin/(−)-epicatechin derivatives. MS/MS data for the spots at 1.122 and 1.156 1/K_0_ were similar indicating a *γ*-configuration. However, it was not possible to obtain MS/MS fragmentation data for the less intense spot at 1.024 1/K_0_ which may also correspond to a protomer or an isomer.

**Peaks 22–24** corresponded to the (−)-epicatechin model (Table S2). MS/MS data from the peaks 22 and 24 indicated a *γ*-configuration, but it was not possible to determine the configuration for the spot at 1.124 1/K_0_ of peak 24 and peak 23. It is important to note that peak 22 was not well-visible in the chromatogram, but well-detected in the heat map.

As described in the method development section, di, tri, and tetramers were generally separated in specific mobility regions (Figure S2) which allowed obtaining, in general, MS spectra of good quality because they can be obtained only for a specific spot. However, it was possible to find masses corresponding to trimers in the zone where the dimers were more present. Kawazoe and co-workers [33] described a cyclic epicatechin trimer produced by oxidative coupling. This type of compound may show a lower mobility than the non-cyclic trimers in the TIMS. Residual ions, mainly in the range of *m*/*z* 863–865, were observed in the zone where the dimers were studied, forming large regions in the heat map (Figure S2). In this work, around 100 spots containing dimeric compounds were analyzed in the flavan-3-ols oxidation models and only in around 50 spots the masses of oligomers were observed in the MS spectrum, indicating a coelution. Among them, 33 spots contained oligomers as monocharged ions with a relative abundance lower than 20%, and only three spots showed an ion at *m*/*z* 863 at 100%. They corresponded to two spots of peak 30 and to the spot of peak 31. In these cases, the relative abundance of the ions at *m*/*z* 577 ranged from 57 to 98% (Table S2).

We investigated if the ions at *m*/*z* 577 of the spots of peaks 30 and 31 were fragments of the *m*/*z* 863 because of the great abundance of this last ion. A heat map was generated using a mass range of *m*/*z* 863.1–863.2 (data not shown). It was observed that the signals of the ion at *m*/*z* 863 covered a larger region of this heat map, where the spots of the peaks 30 and 31 were present, which can explain the presence of the ion at *m*/*z* 863 in all those spots of peak 30 (mainly in the spots at 1.033 and 1.055 1/K_0_) and 31. If the ion at *m*/*z* 863 had been fragmented to generate the ions at *m*/*z* 577 of the peaks 30 and 31, a vast undefined region in the heat map corresponding to the fragment at *m*/*z* 577 would likely be detected, as observed for the ion at *m*/*z* 863. Instead, the ions at *m*/*z* 577 of peaks 30 and 31 were detected as small and well-defined spots. In addition, the MS/MS fragment at *m*/*z* 577 from a dehydrotricatechin at *m*/*z* 863 are not so frequent. Verloop and co-workers [14] detected this fragment in only one of the twelve dehydrotricatechins at *m*/*z* 863 produced from (−)-epicatechin and He and co-workers [13] did not report this fragment in any of the four dehydrotricatechins derived from (−)-epicatechin and/or (+)-catechin. Therefore, the ions at *m*/*z* 577 of the spots of peak 30 and 31 most likely corresponded to dehydrodicatechins rather than fragment of the ion at *m*/*z* 863.

### 2.6. Analysis of Grape Seed Extracts

**Figure 8:**
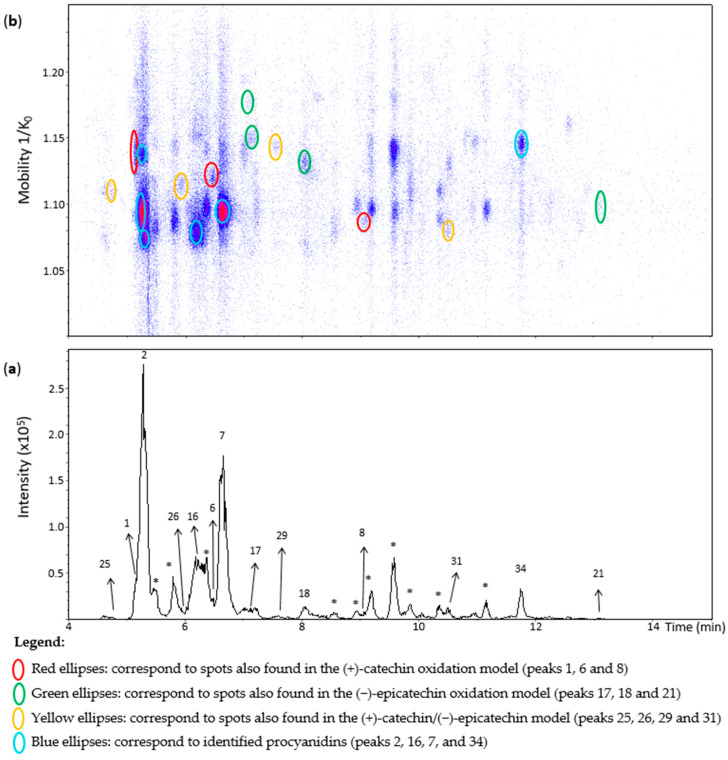
UHPLC−ESI (−)−TIMS−QTOF−MS/MS analysis of grape seed extract. (**a**) EIC from the mass range of *m*/*z* 577.1–577.2; (**b**) heat map showing the mobility versus Rt for the mass range of *m*/*z* 577.1–577.2. Full details on peak numbers are available in Table S2. * Indicates peaks showing an MS/MS fragmentation pattern that corresponds to dimeric procyanidin compounds. Ellipses on the heat map mark the main spots.

Figure 8 shows an EIC of a grape seed extract from an *m*/*z* 577.1–577.2 range. **Peak 1** showed one spot in the heat map (Figure 8) that was annotated as the corresponding spot at 1.129 1/K_0_ in the (+)-catechin model (Table S2). The other two minor spots observed in the (+)-catechin model were not observed in the extract likely due to their low concentrations. Similarly, compounds of the spots of **peaks 25**, **29, and 18** (Figure 8) were annotated as *β*-configuration compounds from the comparison with the data of the oxidation models. On the other hand, **peak 21** corresponded to a compound of *ε*-configuration based on (−)-epicatechin units (Table S2). Despite being barely visible on the chromatogram, **peak 17** generated two misaligned spots (Figure 8). Their MS/MS spectra showed low intensities with mainly the fragment at *m*/*z* 425.08. Even so, they may correspond to two different dehydrodicatechins of *β*-configuration, as in the oxidation models.

**Peak 31** showed a spot at 1.080 1/K_0_ (Figure 8) as in the (+)-catechin/(−)-epicatechin model. Nevertheless, this spot showed the additional MS/MS fragments at *m*/*z* 451, 407, and 289 that are typical for B-type procyanidins (Table S2). Particularly, the fragment at *m*/*z* 451 is specific for these native dimers [19]. Therefore, this peak may correspond to a mixture of dehydrodicatechin of *β*-configuration with a procyanidin. In the same way, **peak 26 and 8**, even if not well-observed in the EIC, could be detected in the heat map and showed an additional fragment at *m*/*z* 451 for peak 26 and *m*/*z* 451, 407, and 289 for peak 8 (Table S2) which may also correspond to the same kind of mixture, containing a procyanidin. The dehydrodicatechin of peak 26 was annotated as in the (+)-catechin/(−)-epicatechin model, and peak 8 as in the (+)-catechin model. Additionally, the ions at *m*/*z* 577.1343 and *m*/*z* 577.1334 of peak 26 an 8 had relative abundances of 53% and 57% in the MS spectra, respectively. The base peak of these spectra corresponded to the ions at *m*/*z* 865.1966 and *m*/*z* 865.1963, respectively, corresponding to flavan-3-ol trimers having the formula [C_45_H_37_O_18_]^−^ (error < 2.7 ppm). Finally, **peak 6** may also correspond to a mixture of a dehydrodicatechin of *β*-configuration, as observed for the (+)-catechin/(−)-epicatechin model, with a procyanidin type compound taking into account the MS/MS data. In these cases, described for peaks 31, 26, 8, and 6, the corresponding dehydrodicatechins were not separated from procyanidins by IMS. Eleven dehydrodicatechins were tentatively identified in this extract. They can be formed by enzymatic oxidation or autoxidation [13,15,19].

Deshaies and co-workers [15] studied different grape seed extracts using an UHPLC-Q-Orbitrap system operated at the positive ion mode. They identified three dehydrodicatechins even if at low abundances corresponding to the N6, N7, and N8 standards. These compounds were not detected in the present work possibly because of the differences among the samples. The present work using an UHPLC−ESI−TIMS−QTOF−MS/MS system was also able to detect several compounds at low concentrations, probably due to the combination of the negative ion mode with TIMS. Indeed, selectivity and sensitivity were higher in negative mode than in positive mode for flavan-3-ols in another work [19], and they can be improved by the use of the IMS that increases the signal-to-noise ratio [21] by eliminating interfering compounds.

**Peaks 2**, **16**, **7, and 34** were identified using standards (Table S2) such as procyanidins B1, B3, B4, B2, and B5 that were already reported for grape seed extracts [18,34].

**Figure 9:**
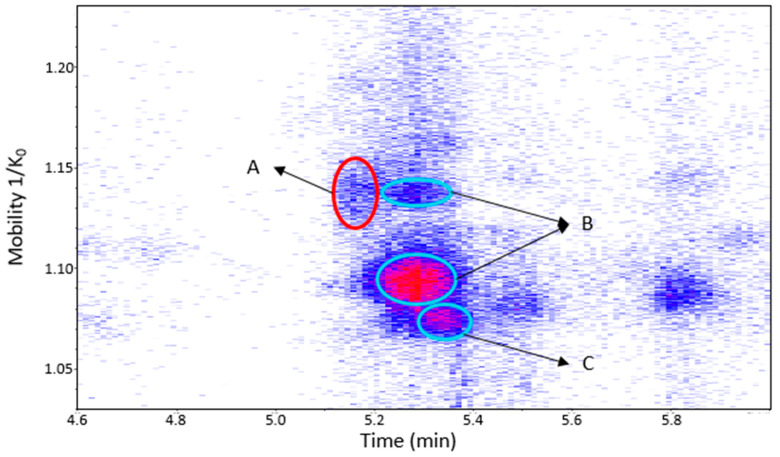
UHPLC−ESI (−)−TIMS−QTOF−MS/MS analysis of grape seed extract: heat map showing the mobility versus Rt for the mass range of *m*/*z* 577.1–577.2. Ellipses on the heat map mark the main spots. A: dehydrodicatechin of *β*-configuration formed from (+)-catechins. B: protomers of procyanidin B1. C: procyanidin B3. Full details are available on Table S2 in peaks 1 and 2 of grape seed extract.

Procyanidin B1 and B3 and the dehydrodicatechin of peak 1 were separated in the mobility dimension (Figure 9). Procyanidin B1 and B3 showed two spots and one spot, respectively (Table S2). These last two compounds are often (partly) coeluted in chromatography [19] and could not be distinguished on the basis of LC-MS/MS data alone in the present study. Li and co-workers [28] reported a slight mobility separation of these compounds with Rt of 12.3 min and drift time of 26.05 milliseconds for procyanidin B1, and 13 min and 25.79 milliseconds for procyanidin B3. Guyot and co-workers and Sun and co-workers [10,19] also described a chromatographic coelution of procyanidin B3 with a dehydrodicatechin B derived from (+)-catechin. These separations illustrate the additional gain that the TIMS provides for the analysis of these compounds.

Dehydrodicatechin of peak 34 of the (+)-catechin/(−)-epicatechin model solution was not detected in this extract, but if it is coeluted with procyanidin B5 in another matrix, they would be easily distinguished by their different mobility values (Table S2).

Several other chromatographic peaks marked with asterisks were observed in Figure 8. Their MS/MS fragmentation pattern were similar to that of B-type procyanidins, except for those eluted at 8.9, 9.2, 9.6, and 11.2 min which showed additional abundant fragment ions at *m*/*z* 245, 330, 331, and 467. These compounds were not the focus in this work. They may be other procyanidins, other types of flavan-3-ols isomers, a mixture of compounds or fragments of procyanidin oligomers. Finally, no dehydrodicatechin A was detected in the grape seed extract.

The different studied compounds were observed as spots having specific mobilities and Rt values in a complex heat map which improved their detection. A similar approach using a mobility-*m*/*z* graph was also used as a tool for characterization [21]. For the purpose of a more detailed characterization including the study of the gas-phase behavior of molecules and their (de)protonation site isomers, the use of molecular modelling along with theoretical and experimental collision cross-section values can be used [35].

## 3. Materials and Methods

### 3.1. Materials

Standards of (+)-catechin (≥98%) and (−)-epicatechin (≥90%) were purchased from Sigma-Aldrich (Steinheim, Germany), procyanidin B1 (≥90%) and procyanidin B2 (≥90%) were purchased from Extrasynthese (Genay, France), procyanidin B3 (≥99%) was acquired from Phytolab (Vestenbergsgreuth, Germany) and procyanidin B4 (≥95%) and procyanidin B5 (≥95%) were acquired from TransMIT PlantMetaChem (Giessen, Germany), and L-ascorbic acid from Sigma-Aldrich (Steinheim, Germany). LC−MS-grade acetonitrile, LC−MS-grade methanol (MeOH), LC−MS-grade formic acid, from Biosolve (Valkenswaard, The Netherlands) and purified water obtained from a Milli-Q water Millipore system (Bedford, MA, USA) were used for the UPLC runs. A total of 500 mg Sep-Pak C18 solid phase extraction (SPE) cartridges were purchased from Waters Corporation (Milford, MA, USA). Reference compounds or fractions N1 to N8 were used (Figure 1).

### 3.2. Reference Compound Solutions

Around 700 µL of individual reference compounds N1 to N8 of unknown concentration in MeOH/H_2_O/HCOOH (49.5/49.5/1, *v*/*v*/*v*) were evaporated under a reduced pressure atmosphere at 30 °C using a GenVac vacuum evaporator (Ipswitch, UK) for approximately 30 min. A total of 150 µL of MeOH/H_2_O/HCOOH (12/87/1) were added to the residual solution. Standards of procyanidins B1-B5 were weighed and individual solutions of 10 µM containing MeOH/H_2_O/HCOOH (12/87/1) were prepared. All these reference compounds were then individually injected into the UPLC system.

### 3.3. Extraction of Grape Polyphenoloxidase

Grape PPO was extracted as described by Cheynier and co-workers [36]. About 200 g of Grenache blanc grapes from INRAE experimental vineyard at Pech Rouge (Gruissan, France) were pressed and mixed with 100 mL of 1.5 M acetate buffer (pH 5) and 1 g of ascorbic acid. The mixture was then filtered (0.5 mm porosity) and centrifuged at 9000 rpm at 4 °C for 10 min. The deposit containing PPO was washed with 80% acetone to remove chlorophylls and finally dried under nitrogen gas flow. During the extraction process, the temperature was kept below 5 °C and the atmosphere was reduced in oxygen as much as possible by pressing the grapes and filtering under vacuum and saturating the centrifuge tubes with nitrogen gas. The dried enzyme extract was stored at −18 °C until use.

### 3.4. Preparation of Oxidation Dimers of Flavan-3-ols

Oxidation procedure as based on Guyot and co-workers [10]. Dried crude grape PPO extract was suspended in milli-Q water (pH~5) to a concentration of 6 mg/mL and sonicated for 2 min at room temperature. A total of three aliquots of this mixture were transferred into three test tubes and used to solubilize (−)-epicatechin, (+)-catechin, and a mixture of the two compounds, respectively, to a final concentration of 6 mM for each compound, including each compound of the mixture of the two flavan-3-ols. After a further 2 min sonication, the uncapped tubes were incubated with shaking for one hour at room temperature. Afterwards, the tubes were centrifuged at 10,000 rpm and at 4 °C and the supernatants were recovered.

The supernatants underwent an SPE using a glass vacuum manifold and C18 SPE cartridges based on Ferreira-Lima and co-workers [37] for the elimination of interferents such as residual salts of the buffer solution used for the PPO extraction as well as the PPO. Cartridges were conditioned with 12 mL of MeOH and 12 mL of H_2_O/HCOOH (99/1). Cartridges were then loaded with 1 mL of each sample. Interferents were eliminated by the elution of 12 mL of H_2_O/HCOOH (99/1). After that, fraction of interest containing the flavan-3-ol oxidation products was obtained by elution with 4mL of MeOH/H_2_O/HCOOH (70/29/1). This fraction was evaporated under a reduced pressure atmosphere at 30 °C using a GeneVac evaporator (Ipswich, UK), and stored at −21 °C until analysis. On the day of analysis, the fraction was solubilized with 1 mL of MeOH/H_2_O/HCOOH (12/87/1) and injected into the UPLC system in duplicate.

### 3.5. Preparation of Grape Seed Samples

A dried grape seed extract was kindly provided by Groupe Grap’Sup (France). About 50 mg of dried grape seed extract were solubilized with MeOH/H_2_O/HCOOH (12/87/1) in a volumetric flask of 5 mL. The mixture was sonicated for 5 min at room temperature, and then centrifuged at 16,000 rpm for 10 min at room temperature. The supernatant was recovered and injected into the UPLC system in duplicate.

### 3.6. UHPLC−ESI−TIMS−QTOF−MS/MS Analyses

The chromatographic analyses were performed using an Acquity UPLC system from Waters Corporation (Milford, MA) that consisted of an autosampler, a column oven, a PDA detector, and a pump module with a built-in solvent degasser, all piloted by 1.70 Waters Acquity UPLC console and 5.1 Compass HyStar softwares. The column used was a Waters Acquity UPLC^®^ HSS T3 C18 column, 1.8 µm, 100 mm × 1.0 mm ID (Wexford, Ireland) equipped with an UltraShield UHPLC pre-column filter, 0.2 µm, from Restek Corporation (Lisses, France). A total of 2 µL of samples were injected and the mobile phase was eluted with a constant flow rate of 220 µL/min with the eluents being (A) 1% formic acid in water and (B) acetonitrile/water/formic acid (80/19/1). The following gradient was used: 0–1.5 min: 2% B; 1.5–4.5 min: 2–12% B; 4.5–7 min: 12% B; 7–12 min: 12–24% B; 12–15 min: 24–48% B; 15–16 min: 48–60% B; 16–17 min: 60–100% B; 17–19 min: 100% B; 19–20 min: 100–2% B; and 20–24 min: 2% B. Column and sample temperatures were kept at 35 and 10 °C, respectively.

The UHPLC system was hyphenated with a Bruker timsTOF system (Bremen, Germany) that consisted of an ESI source, a TIMS analyzer, a hybrid quadrupole, and a time-of-flight mass spectrometer. All parameters were controlled through the 6.2 OtofControl software (Bruker). The operating conditions of the ESI source in negative ion mode were 500 V end plate offset, 3500 V capillary voltage, 40 psi nebulizer pressure, and 10 L/min dry N_2_ gas at a temperature of 200 °C. For the TIMS analyzer, the funnel 1 RF, funnel 2 RF, multipole RF and deflection delta were set at 250 Vpp, 250 Vpp, 200 Vpp, and −70 V, respectively. The 1/K_0_ values ranged from 1 to 1.25 V.s/cm^2^, using a ramp time of 150 ms and the spectra rate of 6.45 Hz. In addition, the accumulation time was adjusted to 75 ms, corresponding to a duty cycle of 50%.

MS experiments were performed in a full scan mode and in an auto-MS/MS mode (MS and MS/MS experiments are performed alternately) for a mass range of *m*/*z* 150–1500. MS/MS experiments were performed to ions in the mass range of *m*/*z* 570–580. Collision-induced dissociation was set to 27.3 eV.

Before each analysis series, an external calibration of the equipment was performed for mass accuracy using a solution of sodium formate clusters prepared according to the manufacturer’s guidelines. Another external calibration was carried out using a tuning mix solution from Agilent technologies (Santa Clara, CA, USA) for ion mobility accuracy. In this case, a minimum of three points were used for a linear calibration. Additionally, 20 µL of the sodium formate solution was injected via a syringe, a sample loop and a 6-ways divert valve into the mass spectrometer at the beginning of each UHPLC gradient for internal sample calibration. The 5.3 data analysis software (Bruker) was used for data processing.

## 4. Conclusions

An UHPLC-(−)ESI-TIMS-QTOF-MS/MS method was developed for the analysis of dehydrodicatechins. Oxidation models using flavan-3-ols and a grape PPO extract were used for the production of dehydrodicatechins. More than 30 dehydrodicatechins derived from (+)-catechin, (−)-epicatechin, and both (+)-catechin and (−)-epicatechin were tentatively annotated, without counting the possible protomers or other isomers, and they had different interflavanic configuration (*β*, *γ* and *ε*-configuration). At least 11 dehydrodicatechins and procyanidins B1-B5 were (tentatively) identified in a grape seed extract. Among the compounds, 4, 4, and 3 dehydrodicatechins were based on both (+)-catechin and (−)-epicatechin, (−)-epicatechin, and (+)-catechin, respectively. All of them were dehydrodicatechins with *β*-configuration, except for one compound that was of *ε*-configuration. UHPLC−TIMS−MS allowed a good graphic visualization of interest compounds in a complex matrix. It also allowed mobility separation of isomers (partly) coeluted by chromatography that would hardly be distinguished by LC-MS/MS alone. To the best of our knowledge, this is the first time that the dehydrodicatechins produced from catechol-type flavan-3-ols were studied by IMS, which was already applied to investigate other similar compounds such as theasinensins produced by oxidation of pyrogallol-type flavan-3-ols [27]. This method can be used or adapted for other food or natural products containing dimers of different flavan-3-ols units.

## Data Availability

The data presented in this study are available in supplementary material.

## Supplementary Materials

The following supporting information can be downloaded at: https://www.mdpi.com/article/10.3390/molecules27134176/s1, Figure S1: MS/MS spectra of the reference compound N2, N6, N8 and procyanidin B3; Figure S2: UHPLC-ESI (−)-TIMS-QTOF-MS/MS analysis of a mixture of flavan-3-ols oxidation products with procyanidins B1 and B3; Figure S3: UHPLC-ESI (−)-TIMS-QTOF-MS/MS analysis of reference compound N2, a dehydrodicatechin with *β*-configuration based on (+)-catechins; Figure S4: Partial MS/MS fragmentation pathway of N2 standard, a dehydrodicatechin with *β*-configuration, at *m*/*z* 577.1343 ([C_30_H_25_O_12_]^−^) and 1.099 1/K_0_; Figure S5: Partial MS/MS fragmentation pathway of N3 standard, a dehydrodicatechin with *ε*-configuration, at *m*/*z* 577.1343 ([C_30_H_25_O_12_]^−^) and 1.076 1/K_0_; Figure S6: Partial MS/MS fragmentation pathway of N4 standard, a dehydrodicatechin with *β*-configuration, at *m*/*z* 577.1341 ([C_30_H_25_O_12_]^−^) and 1.084 1/K_0_; Figure S7: Partial MS/MS fragmentation pathway of N6 standard, a dehydrodicatechin with *ε*-configuration, at *m*/*z* 577.1361 ([C_30_H_25_O_12_]^−^) and 1.092 1/K_0_; Figure S8: Partial MS/MS fragmentation pathway of N8 standard, a dehydrodicatechin with *γ*-configuration, at *m*/*z* 575.1197 ([C_30_H_23_O_12_]^−^) and 1.164 1/K_0_; Figure S9: Partial MS/MS fragmentation pathway of procyanidin B3 at *m*/*z* 577.1353 ([C_30_H_25_O_12_]^−^) and 1.091 1/K_0_; Figure S10: Mass spectra of N1a compounds having different mobilities. Table S1: UHPLC-ESI-TIMS-QTOF-MS/MS data of reference compounds of dehydrodicatechins formed from (+)-catechins (N1–N8) and dimeric procyanidins; and Table S2: Dehydrodicatechins tentatively identified in the flavan-3-ols oxidation models and grape seed extract.

## Sample Availability

Samples of the compounds are not available from the authors.
