# Supplementary material for: Improved Analysis of Isomeric Polyphenol Dimers Using the 4th Dimension of Trapped Ion Mobility Spectrometry—Mass Spectrometry
Source: Molecules. 2022 Jun 29;27(13):4176. doi: 10.3390/molecules27134176 (PMC9268536; doi:10.3390/molecules27134176)
Supplement: Supplementary file 1 [file molecules-27-04176-s001.zip › molecules-1789436-supplementary.pdf]

Supplementary material

Improved analysis of isomeric polyphenol dimers using the 4th dimension of trapped ion mobility spectrometry - mass spectrometry

Aécio L. de S. Dias[1,2] ; Arnaud Verbaere[1,2] ; Emmanuelle Meudec[1,2] ; Stacy Deshaies[1] ; Cédric Saucier[1] ; Véronique Cheynier[1,2] ; Nicolas Sommerer[1,2]*

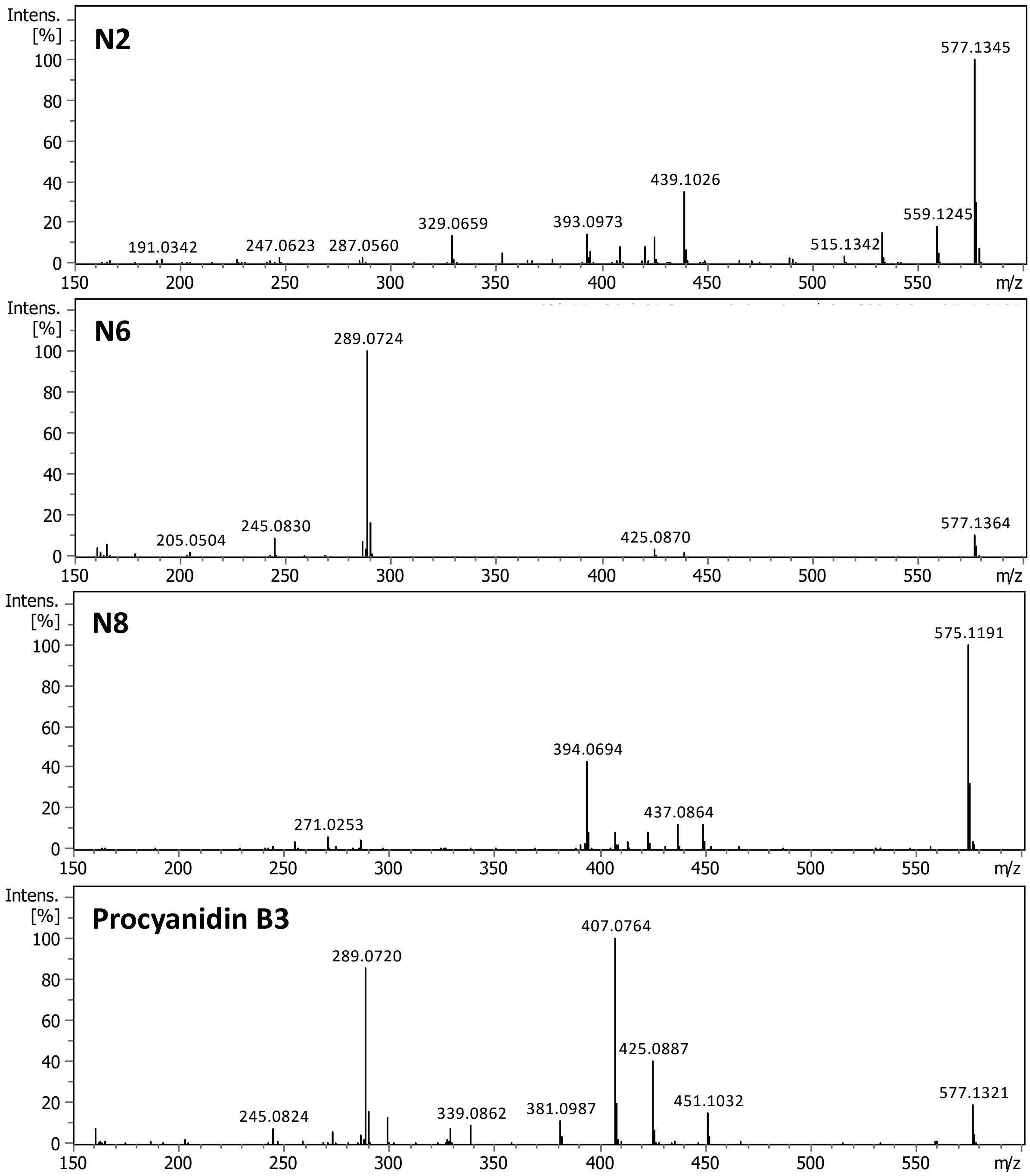

Figure S1 : MS/MS spectra of the reference compound N2, N6, N8 and procyanidin B3. N2, N6 and N8 compounds correspond to dehydrodicatechins with β, ε and γ-configuration, respectively

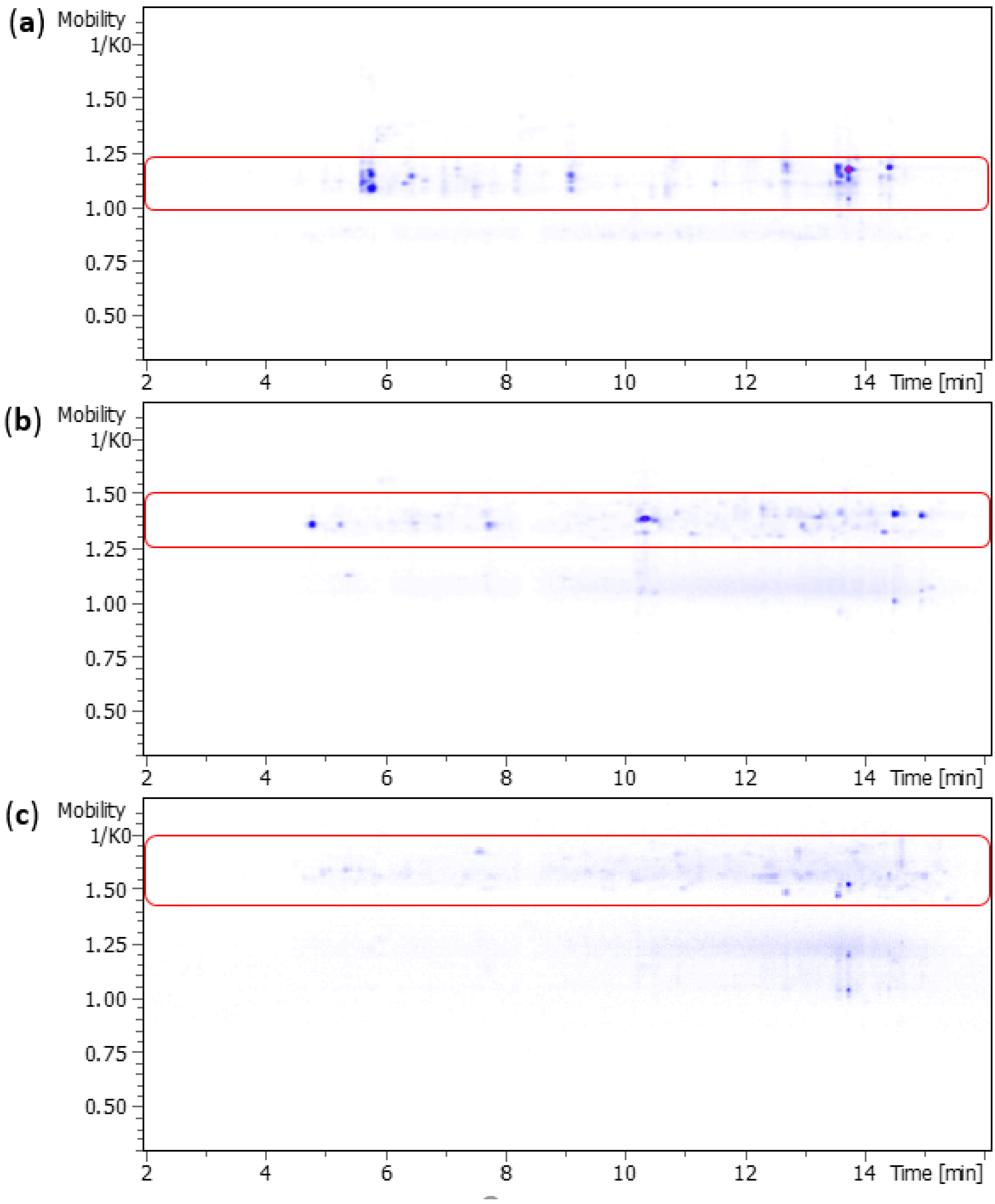

Figure S2 : UHPLC–ESI (−)–TIMS–QTOF–MS/MS analysis of a mixture of flavan-3-ols oxidation products with procyanidins B1 and B3: (a) Heat map showing the mobility versus Rt for the mass ranges of *m/z* 577.1-577.2 ; (b) *m/z* 861.1-865.3 and (c) *m/z* 1151.2-1153.3

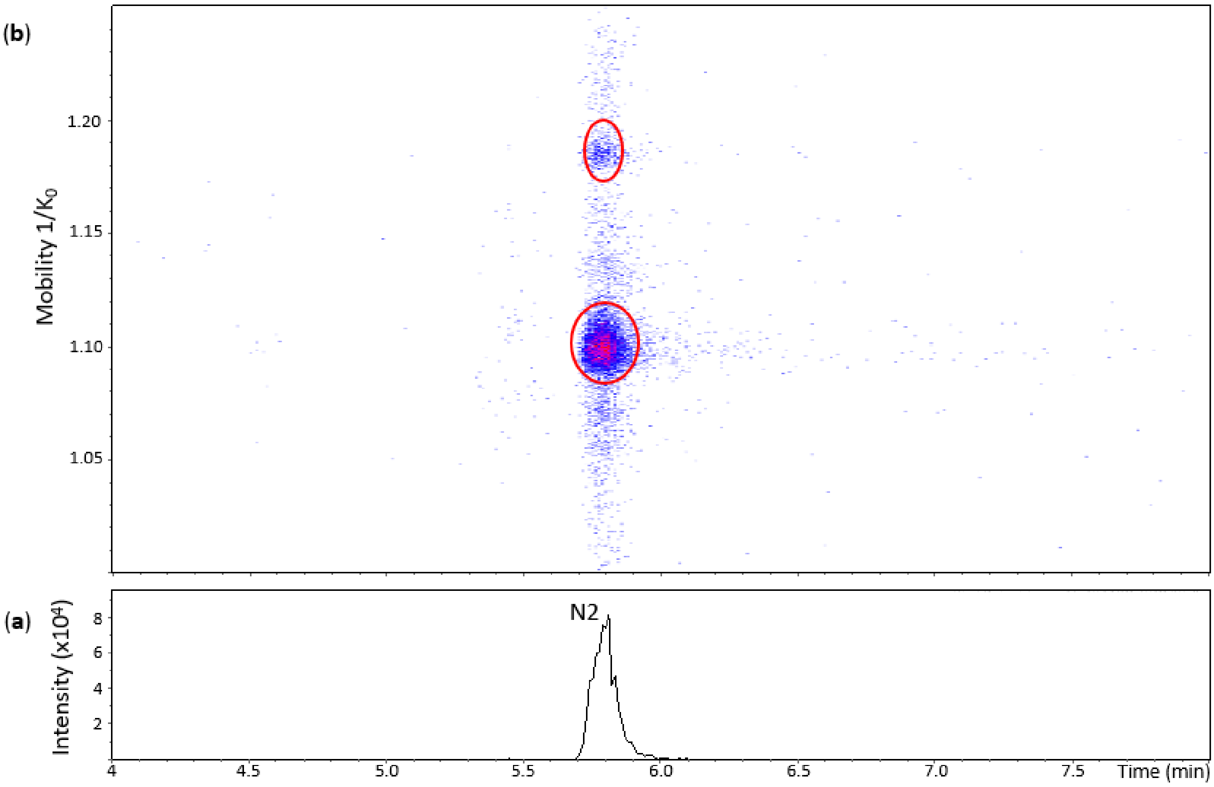

Figure S3 : UHPLC−ESI (−)−TIMS−QTOF−MS/MS analysis of reference compound N2, a dehydrodicatechin with *β*-configuration based on (+)-catechins. (a) EIC from the mass range of *m/z* 575.1-575.2 ; (b) Heat map showing the mobility versus Rt for the mass range of *m/z* 575.1-575.2. Full details on the compound are available in Table S2. Ellipses on the heat map mark the main spots

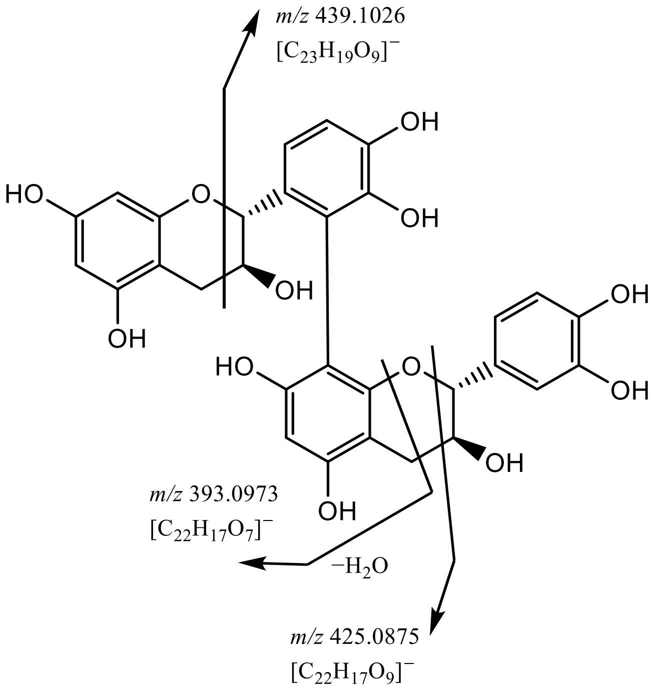

Figure S4 : Partial MS/MS fragmentation pathway of N2 standard, a dehydrodicatechin with *β*-configuration, at *m/z* 577.1343 ([C30H25O12]-) and 1.099 1/K0. Full details on the compound are available in Table S1

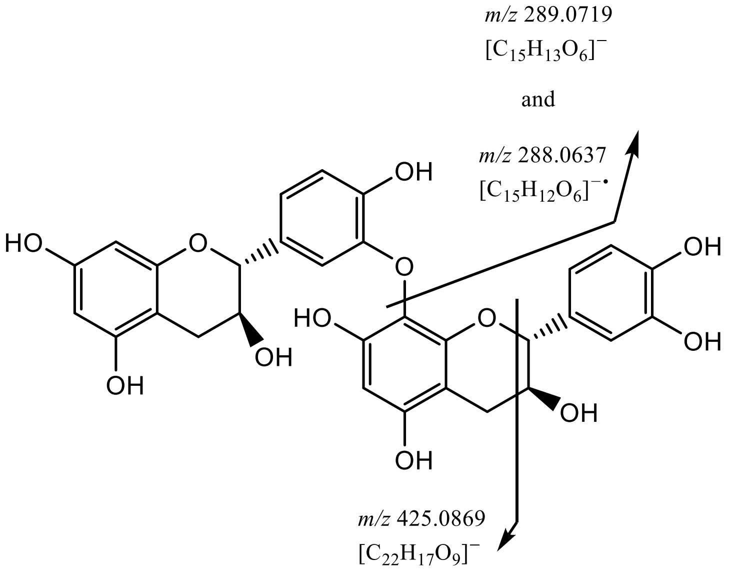

Figure S5 : Partial MS/MS fragmentation pathway of N3 standard, a dehydrodicatechin with *ε*-configuration, at *m/z* 577.1343 ([C30H25O12]⁻) and 1.076 1/K0. Full details on the compound are available in Table S1

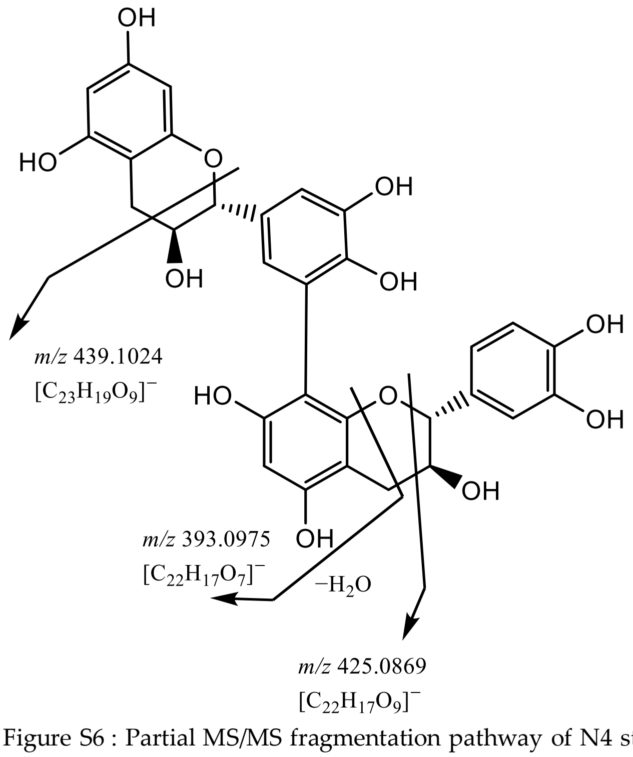

Figure S6 : Partial MS/MS fragmentation pathway of N4 standard, a dehydrodicatechin with *β*-configuration, at *m/z* 577.1341 ([C30H25O12]⁻) and 1.084 1/K0. This standard corresponds to a mixture of enantiomers. Full details on the compound are available in Table S1

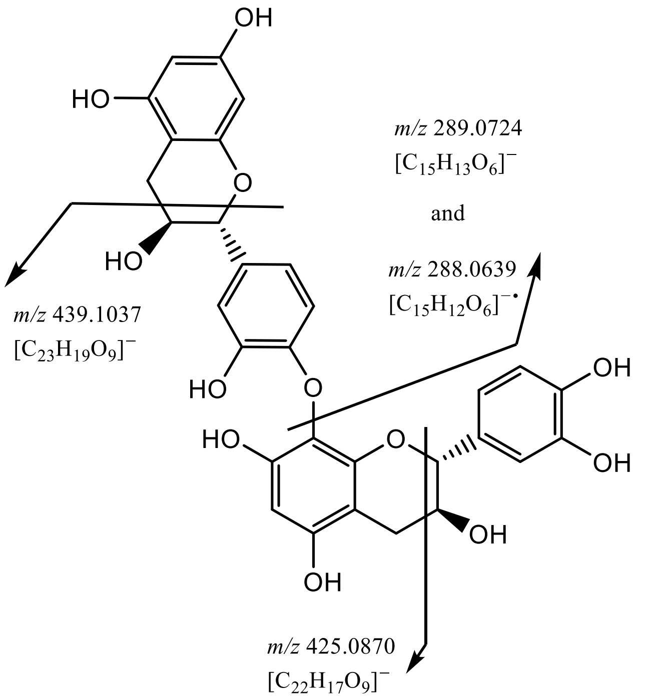

Figure S7 : Partial MS/MS fragmentation pathway of N6 standard, a dehydrodicatechin with ε-configuration, at *m*/*z* 577.1361 ([C30H25O12]⁻) and 1.092 1/K0. Full details on the compound are available in Table S1

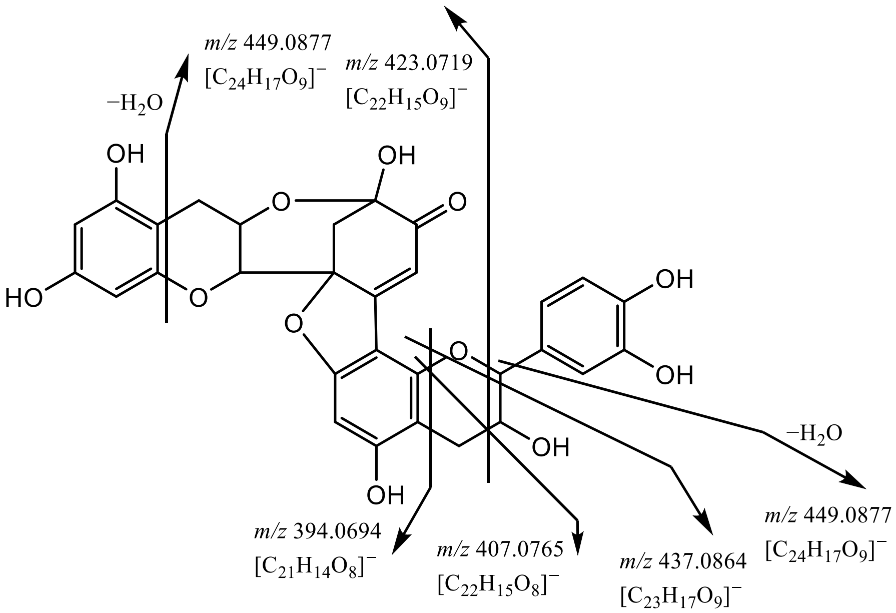

Figure S8 : Partial MS/MS fragmentation pathway of N8 standard, a dehydrodicatechin with γ-configuration, at *m*/*z* 575.1197 ([C30H23O12]⁻) and 1.164 1/K0. Full details on the compound are available in Table S1

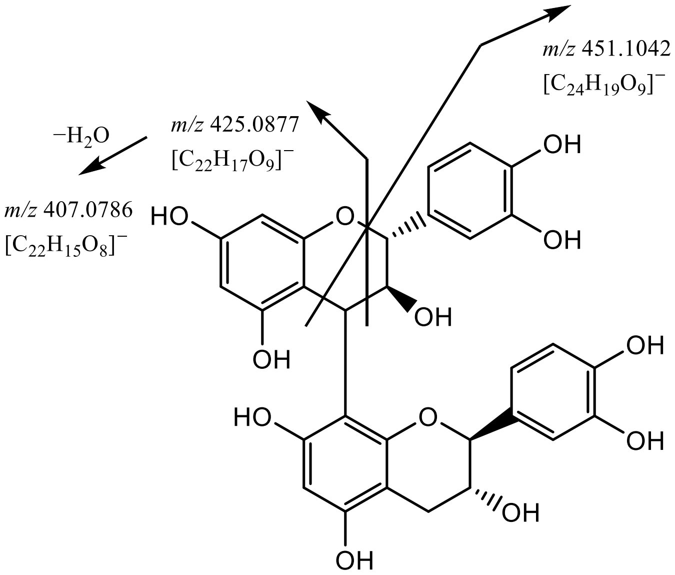

Figure S9 : Partial partial MS/MS fragmentation pathway of procyanidin B3 at *m*/*z* 577.1353 ([C30H25O12]⁻) and 1.091 1/K0. Full details on the compound are available in Table S1

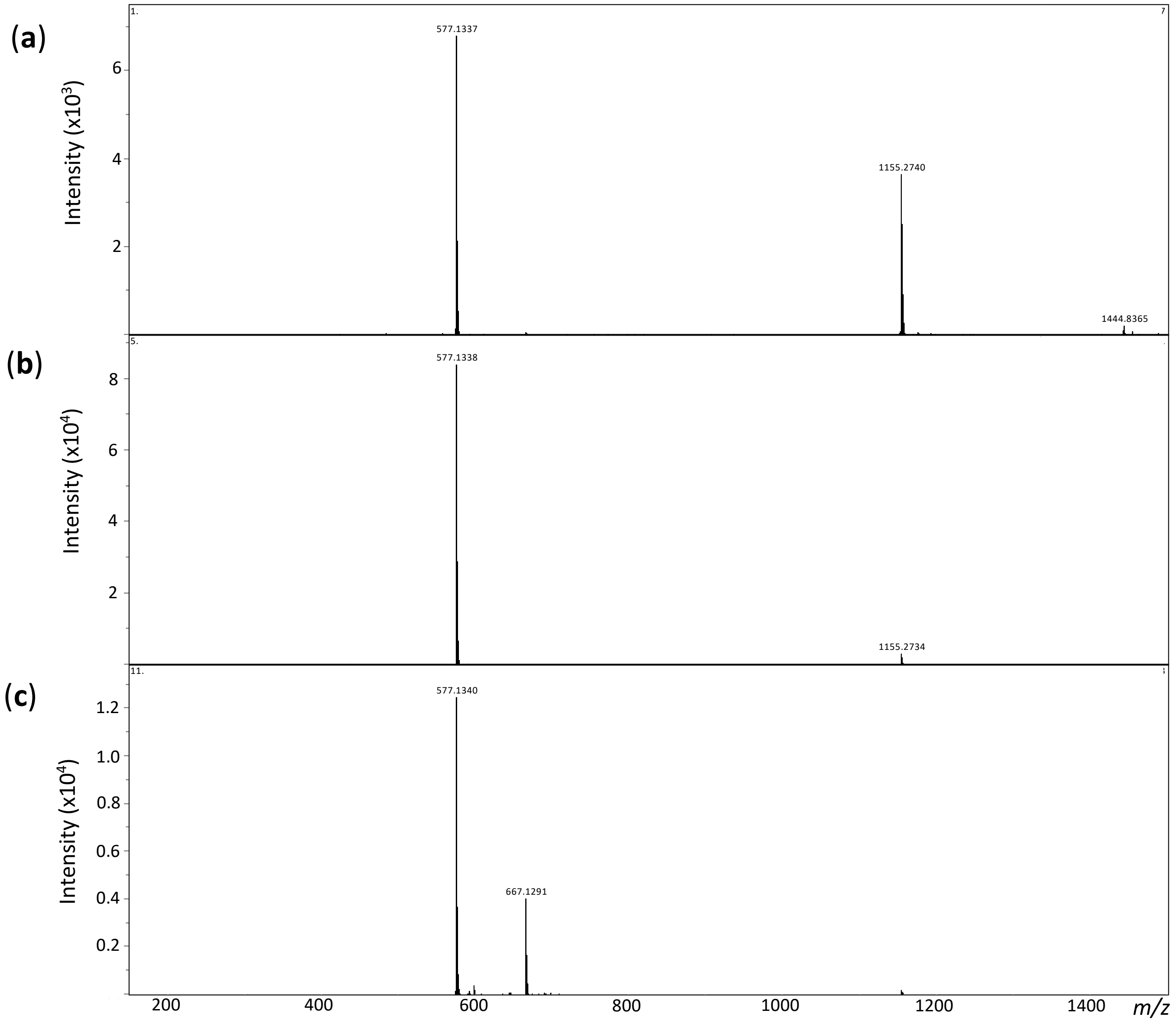

Figure S10 : Mass spectra of N1a compounds having different mobilities (a) at 1.064 $1/K_0$, (b) 1.128 $1/K_0$ and (c) 1.191 $1/K_0$. Full details on the compounds are available in Table S1

Table S1 : UHPLC−ESI−TIMS−QTOF−MS/MS data of reference compounds of dehydrodicatechins formed from (+)-catechins (N1-N8) and dimeric procyanidins

Reference compound [a]	Rt (min)	Mobility ($1/K_0$)	Ion (*m/z*) (relative abundance %) [c,d]	Ion identity proposal	MS/MS fragments of $[M-H]^-$ (relative abundance %)	Annotation [e, f, g]
N1a	5.2	1.064	577.1337 (100)<br>1155.2740 (54)	$[M-H]^-$<br>$[2M-H]^-$	**577.1337 (100)**, 559.1260 (6), 533.1452 (11), 439.1024 (23), 425.0881 (14), 393.0973 (16)	C-β_{AB}-C
		1.128 [b]	577.1338 (100)	$[M-H]^-$	**577.1342 (100)**, 559.1224 (5), 533.1455 (10), 439.1022 (45), 425.0872 (14), 393.0979 (17)	C-β_{AB}-C [b]
		1.191	577.1340 (100)<br>667.1291 (32)	$[M-H]^-$<br>$[(M-2H)+2HCOOH-H]^-$	m.d.	
N1b	5.3	1.069	577.1343 (100)<br>1155.2753 (9)	$[M-H]^-$<br>$[2M-H]^-$	**577.1348 (100)**, 559.1259 (7), 533.1435 (9), 439.1027 (25), 425.0873 (19), 393.0970 (18)	C-β_{AB}-C
		1.110	577.1338 (100)<br>667.1299 (6)<br>1155.2745 (70)	$[M-H]^-$<br>$[(M-2H)+2HCOOH-H]^-$<br>$[2M-H]^-$	**577.1340 (100)**, 559.1193 (10), 533.1490 (8), 425.0862 (8), 393.0974 (16)	C-β_{AB}-C
		1.136 [b]	577.1338 (100)	$[M-H]^-$	**577.1340 (100)**, 559.1215 (12), 533.1456 (18), 439.1015 (82), 425.0884 (27), 393.1105 (7)	C-β_{AB}-C [b]
		1.155	577.1339 (100)	$[M-H]^-$	**577.1340 (100)**, 533.1452 (6), 439.1019 (44), 425.0880 (9), 393.0986 (13)	C-β_{AB}-C
		1.187	577.1342 (69)<br>667.1291 (100)	$[M-H]^-$<br>$[(M-2H)+2HCOOH-H]^-$	m.d.	
N1c	5.4	1.113 [b]	577.1344 (100)<br>1155.2757 (13)	$[M-H]^-$<br>$[2M-H]^-$	**577.1343 (100)**, 559.1243 (12), 533.1442 (11), 439.1026 (31), 425.0886 (6), 393.0967 (7)	C-β_{AB}-C [b]
		1.164	577.1341 (100)	$[M-H]^-$	**577.1342 (100)**, 559.1255 (15), 533.1446 (13), 439.1034 (29), 425.0874 (10), 393.0982 (9)	C-β_{AB}-C
N2	5.8	1.099 [b]	577.1343 (100)	$[M-H]^-$	**577.1345 (100)**, 559.1245 (19), 533.1436 (16), 439.1026 (36), 425.0875 (13), 393.0973 (15)	C-β_{AB}-C [b]
		1.185	577.1341 (100)	$[M-H]^-$	m.d.	C-β_{AB}-C
N3	6.7	1.062	577.1338 (100)	$[M-H]^-$	577.1343 (73), **289.0695 (100)**	C-ε_{AB}-C
		1.076 [b]	577.1343 (100)	$[M-H]^-$	577.1381 (38), 425.0869 (71), 289.0719 (44), **288.0637 (100)**	C-ε_{AB}-C [b]
N4	9.2	1.084 [b]	577.1341 (100)	$[M-H]^-$	**577.1342 (100)**, 559.1241 (7), 533.1439 (20), 439.1024 (48), 425.0869 (13), 393.0975 (1)	C-β_{AB}-C [b]
		1.102	577.1339 (100)<br>1155.2754 (14)	$[M-H]^-$<br>$[2M-H]^-$	**577.1342 (100)**, 559.1239 (7), 533.1442 (20), 439.1031 (50), 425.0858 (13), 393.0954 (1)	C-β_{AB}-C [c]
		1.122	577.1337 (100)<br>1155.2755 (13)	$[M-H]^-$<br>$[2M-H]^-$	**577.1343 (100)**, 559.1233 (7), 533.1441 (19), 439.1030 (54), 425.0865 (14), 393.0957 (1)	C-β_{AB}-C [c]
		1.155	577.1338 (100)<br>667.1305 (9)<br>1155.2745 (14)	$[M-H]^-$<br>$[(M-2H)+2HCOOH-H]^-$<br>$[2M-H]^-$	**577.1309 (100)**, 439.1032 (29), 425.0844 (30)	C-β_{AB}-C [c]
N5	10.2	1.195	577.1352 (100)	$[M-H]^-$	**577.1350 (100)**, 559.1264 (5), 533.1452 (17), 439.1031 (45), 425.0880 (22)	C-β_{AB}-C
N6	11.0	1.092	577.1361 (100)	$[M-H]^-$	577.1364 (11), 439.1037 (2), 425.0870 (4), **289.0724 (100)**, 288.0639 (4)	C-ε_{AB}-C
N7	12.2	1.100	575.1196 (100)	$[M-H]^-$	**575.1196 (100)**, 557.1102 (57), 531.1322 (20), 449.0866 (10), 437.0858 (60)	C-γ_{AB}-C/C-δ_{AB}-C
N8	13.3	1.097	575.1195 (100)<br>1151.2453 (15)	$[M-H]^-$<br>$[2M-H]^-$	**575.1213 (100)**, 449.0860 (26)	
		1.164 [b]	575.1197 (100)	$[M-H]^-$	**575.1191 (100)**, 449.0877 (13), 437.0864 (13), 423.0719 (9), 407.0765 (9), 394.0694 (43)	C-γ_{AB}-C [b]

Table S1 : Continued

**Reference compound** [a]	**Rt (min)**	**Mobility ($1/K_0$)**	**Ion (*m/z*) (relative abundance %)** [c,d]	**Ion identity proposal**	**MS/MS fragments of $[M-H]^-$ (relative abundance %)**	**Annotation** [e, f, g]
Procyanidin B1	5.3	1.091 [b]	577.1360 (100)	$[M-H]^-$	577.1326 (12), 559.1216 (3), 451.1030 (14), 425.0879 (23), 407.0782 (74), **289.0721 (100)**	C-β_{AC}-EC
		1.128	577.1352 (100)	$[M-H]^-$	425.0878	C-β_{AC}-EC
			667.1322 (22)	$[(M-2H)+2HCOOH-H]^-$		
Procyanidin B2	6.6	1.091	577.1353 (100)	$[M-H]^-$	577.1364 (15), 451.1042 (16), 425.0877 (34), **407.0786 (100)**, 289.0725 (98)	EC-β_{AC}-EC
Procyanidin B3	5.3	1.067	577.1355 (100)	$[M-H]^-$	577.1321 (20), 559.1283 (2), 451.1032 (15), 425.0887 (41), **407.0764 (100)**, 289.0720 (86)	C-β_{AC}-C
Procyanidin B4	6.2	1.075	577.1342 (100)	$[M-H]^-$	577.1344 (12), 559.1228 (2), 451.1030 (20), 425.0867 (35), **407.0767 (100)**, 289.0719 (91)	EC-β_{AC}-C
Procyanidin B5	11.8	1.140	577.1347 (100)	$[M-H]^-$	577.1369 (27), 533.1418 (3), 451.1032 (27), 425.0864 (52), 407.0766 (92), **289.0724 (100)**	EC-β_{AC}-EC

[a] N1 fraction corresponded to three chromatographic peaks named N1a, N1b and N1c. [b] Correspond to compounds showing the most abundant MS and MS/MS signals among the other compounds of a same chromatographic peak. [c] Only adduct ions showing relative intensity >5% are shown. [d] *m/z* 577 and *m/z* 575 corresponded to $[C30H25O12]^-$ and $[C30H23O12]^-$, respectively ; *m/z* 1155 and *m/z* 1151 corresponded to $[C60H51O24]^-$ and $[C60H47O24]^-$, respectively ; and *m/z* 667 corresponded to $[C32H27O16]^-$ (ppm error <3.2). [e] Structures are shown in Figure 1 for N2, N3, N4, N6 and N8 standards. [f] Dehydrodicatechin nomenclature is described in Verloop and co-workers [1]. [g] When annotation is ambiguous, the different options are separated by "/". **m.d.** : missing data. **C** : (+)-catechin. **EC** : (−)-epicatechin

Table S2 : Dehydrodicatechins tentatively identified in the flavan-3-ols oxidation models and grape seed extract

Sample	Peak No	Rt (min)	Mobility ($1/K_0$)	Ion (*m/z*) (relative abundance %) [b,c]	Ion identity proposal	MS/MS fragments of $[M-H]^-$ (relative abundance %)	Tentative annotation [d, e, f]
(+)-catechin oxidation model	1	5.2	1.062	577.1338 (100)	$[M-H]^-$	**577.1332 (100)**, 439.1026 (7), 425.0869 (19)	C-β_{AB}-C [d]
			1.129 [a]	577.1347 (100)	$[M-H]^-$	**577.1342 (100)**, 559.1226 (5), 533.1459 (10), 439.1036 (40), 425.0879 (18), 393.0979 (14)	C-β_{AB}-C [a,d]
			1.189	577.1342 (100)	$[M-H]^-$	m.d.	
				667.1342 (26)	$[(M-2H)+2HCOOH-H]^-$		
				599.1166 (18)	$[(M-H)-H+Na]^-$		
	2	5.3	1.068	577.1340 (100)	$[M-H]^-$	**577.1342 (100)**, 559.1245 (6), 533.1419 (10), 439.1030 (28), 425.0862 (21), 393.0979 (12)	C-β_{AB}-C [d]
				1155.2739 (8)	$[2M-H]^-$		
			1.111	577.1336 (100)	$[M-H]^-$	577.1370 (99), 559.1289 (22), 533.1499 (10), **439.1037 (100)**, 425.0846 (13), 393.0981 (37)	C-β_{AB}-C [d]
				1155.2759 (22)	$[2M-H]^-$		
			1.135 [a]	577.1343 (100)	$[M-H]^-$	**577.1352 (100)**, 559.1224 (5), 439.1035 (42), 425.0890 (17), 393.0992 (2)	C-β_{AB}-C [a,d]
			1.154	577.1338 (100)	$[M-H]^-$	**577.1328 (100)**, 559.1286 (10), 533.1460 (18), 439.1033 (70), 425.0835 (9), 393.0943 (22)	C-β_{AB}-C [d]
			1.189	577.1346 (65)	$[M-H]^-$	m.d.	
				599.1143 (8)	$[(M-H)-H+Na]^-$		
				667.1302 (100)	$[(M-2H)+2HCOOH-H]^-$		
	3	5.4	1.111 [a]	577.1328 (100)	$[M-H]^-$	**577.1354 (100)**, 439.1004(54)	C-β_{AB}-C [a,d]
			1.165	577.1356 (100)	$[M-H]^-$	m.d.	C-β_{AB}-C [d]
	4	5.5	1.082	577.1342 (100)	$[M-H]^-$	m.d.	
	5	5.8	1.098	577.1346 (100)	$[M-H]^-$	**577.1343 (100)**, 559.1240 (16), 533.1437 (10), 439.1028 (27), 425.0868 (12), 393.0980 (9)	C-β_{AB}-C [d]
			1.130 [a]	577.1341 (100)	$[M-H]^-$	**577.1351 (100)**, 559.1286 (5), 533.1490 (18), 439.1044 (21), 425.0860 (16), 393.0975 (8)	C-β_{AB}-C/epimer of that compound [a]
			1.188	577.1363 (100)	$[M-H]^-$	m.d.	C-β_{AB}-C [d]
	6	6.5	1.125	577.1351 (100)	$[M-H]^-$	**577.1286 (100)**, 559.1223 (2), 533.1499 (13), 439.1020 (23), 425.0867 (9), 393.0961 (17)	C-β_{AB}-C/epimer of that compound
	7	6.7	1.063	577.1342 (100)	$[M-H]^-$	577.1301 (19), 425.0903 (75), **289.0713 (100)**	C-ε_{AB}-C [,d]
			1.080 [a]	577.1343 (100)	$[M-H]^-$	577.1307 (86), 425.0900 (18), 393.0992 (18), **289.0736 (100)**	C-ε_{AB}-C [a,d]
	8	9.2	1.087	577.1340 (100)	$[M-H]^-$	577.1282 (79), 559.1298 (12), 439.1012 (94), **425.0861 (100)**	C-β_{AB}-C [d]
	9	10.1	1.173	577.1344 (100)	$[M-H]^-$	**577.1374 (100)**, 439.1071 (30), 289.0745 (18), 288.0624 (58)	C-ε_{AB}-C/epimer of that compound
	10	10.2	1.201	577.1343 (100)	$[M-H]^-$	**577.1300 (100)**, 559.1220 (15), 533.1447 (16), 439.1043 (37), 425.0860 (22)	C-β_{AB}-C [d]
	11	11	1.097	577.1344 (100)	$[M-H]^-$	577.1334 (15), **289.0715 (100)**, 439.0993 (3%)	C-ε_{AB}-C [d]

Table S2 : Continued

Sample	Peak No	Rt (min)	Mobility ($1/K_0$)	Ion (m/z) (relative abundance %) [b,c]	Ion identity proposal	MS/MS fragments of $[M-H]^-$ (relative abundance %)	Tentative annotation [d, e, f]
(+)-catechin oxidation model	12	12.2	1.104	575.1181 (100)	$[M-H]^-$	**557.1070 (100)**	C-γ_{AB}-C/C-δ_{AB}-C [d]
	13	12.3	1.197	575.1199 (100)	$[M-H]^-$	m.d.	
	14	13.2	1.100	575.1187 (100)	$[M-H]^-$	**575.1198 (100)**, 449.0834 (10), 437.0864 (9), 407.0764 (6), 394.0698 (12)	C-γ_{AB}-C
				1151.2446 (16)	$[2M-H]^-$		
			1.169 [a]	575.1192 (100)	$[M-H]^-$	**575.1189 (100)**, 449.0874 (16), 437.0871 (9), 423.0718 (9), 407.0768 (10), 394.0680 (40)	C-γ_{AB}-C [a,d]
(−)-epicatechin oxidation model	15	4.5	1.109	577.1324 (100)	$[M-H]^-$	**439.1040 (100)**	EC-β_{AB}-EC
				645.1239 (7)	$[(M-H)+Na+HCOO^-]^-$		
			1.158 [a]	577.1328 (100)	$[M-H]^-$	**577.1387 (100)**, 533.1494 (68)	EC-β_{AB}-EC [a]
	16	6.2	1.104	577.1345 (100)	$[M-H]^-$	**577.1358 (100)**, 559.1255 (16), 533.1463 (30), 439.0993 (20), 425.0894 (55), 393.0956 (23)	EC-β_{AB}-EC
	17	7.2	1.073	577.1342 (100)	$[M-H]^-$	**577.1320 (100)**, 559.1224 (6), 439.1096 (6), 425.0882 (14), 393.0930 (9)	EC-β_{AB}-EC
			1.148	577.1347 (100)	$[M-H]^-$	577.1355 (75), 559.1226 (35), **425.0861 (100)**	EC-β_{AB}-EC
			1.175 [a]	577.1349 (100)	$[M-H]^-$	**577.1347 (100)**, 559.1301 (13), 533.1421 (8), 439.1034 (20), 425.0851 (32), 393.0964 (19).	EC-β_{AB}-EC [a]
			1.210	577.1357 (100)	$[M-H]^-$	m.d.	
				599.1165 (92)	$[(M-H)-H+Na]^-$		
				667.1312 (35)	$[(M-2H)+2HCOOH-H]^-$		
	18	8.1	1.068	577.1348 (100)	$[M-H]^-$	**577.1351 (100)**, 559.1203 (4), 533.1465 (12), 439.1018 (35), 425.0879 (19), 393.0966 (19)	EC-β_{AB}-EC
			1.131 [a]	577.1344 (100)	$[M-H]^-$	**577.1334 (100)**, 559.1229 (13), 533.1462 (9), 439.1033 (46), 425.0887 (14), 393.0965 (18)	EC-β_{AB}-EC [a]
	19	10.1	1.052	577.1356 (100)	$[M-H]^-$	577.1380 (17), 425.0891 (29), **289.0717 (100)**	EC-ε_{AB}-EC
			1.077 [a]	577.1350 (100)	$[M-H]^-$	577.1342 (17), 425.0869 (7), **289.0710 (100)**	EC-ε_{AB}-EC [a]
	20	12.2	1.100	577.1360 (100)	$[M-H]^-$	**577.1365 (100)**, 533.1474 (21), 439.1008 (64)	EC-β_{AB}-EC
			1.196 [a]	577.1347 (100)	$[M-H]^-$	**577.1364 (100)**, 533.1404 (8), 439.1031 (24), 425.0836 (13)	EC-β_{AB}-EC [a]
	21	13.1	1.098	577.1352 (100)	$[M-H]^-$	577.1339 (17), 425.0849 (2), 289.0721 (100)	EC-ε_{AB}-EC
	22	13.6	1.207	575.1200 (100)	$[M-H]^-$	**575.1192 (100)**, 449.0918 (6), 437.0900 (2), 407.0747 (3)	EC-γ_{AB}-EC/EC-δ_{AB}-EC
	23	14.1	1.044	575.1180 (100)	$[M-H]^-$	**575.1215 (100)**, 437.0867 (14), 407.0747 (9)	EC-γ_{AB}-EC/EC-δ_{AB}-EC
			1.095	575.1196 (100)	$[M-H]^-$	**575.1189 (100)**, 449.0863 (37), 407.0811 (10)	EC-γ_{AB}-EC/EC-δ_{AB}-EC
			1.123 [a]	575.1194 (100)	$[M-H]^-$	**575.1217 (100)**, 449.0857 (71), 437.0867 (20)	EC-γ_{AB}-EC/EC-δ_{AB}-EC [a]
			1.190	575.1187 (100)	$[M-H]^-$	m.d.	
				643.1068 (66)	$[(M-H)+Na+HCOO^-]^-$		
	24	14.2	1.119	575.1183 (100)	$[M-H]^-$	**575.1190 (100)**, 449.0908 (4), 437.0906 (8), 407.0737 (11), 394.0698 (12)	EC-γ_{AB}-EC
			1.168 [a]	575.1192 (100)	$[M-H]^-$	**575.1195 (100)**, 449.0866 (12), 437.0876 (7), 423.0719 (7), 407.0767 (6), 394.0687 (17)	EC-γ_{AB}-EC [a]

Table S2 : Continued

Sample	Peak No	Rt (min)	Mobility ($1/K_0$)	Ion (*m/z*) (relative abundance %) [b, c]	Ion identity proposal	MS/MS fragments of $[M-H]^-$ (relative abundance %)	Tentative annotation [d, e, f]
(+)-catechin/(−)-epicatechin oxidation model	15	4.5	1.110	577.1341 (100)<br>645.1259 (6)	$[M-H]^-$<br>$[(M-H)+Na+HCOO^-]^-$	577.1265 (90), **559.1276 (100)**	EC-β_{AB}-EC
			1.157 [a]	577.1327 (100)	$[M-H]^-$	**577.1298 (100)**, 439.1029 (63)	EC-β_{AB}-EC [a]
	25	4.8	1.108	577.1327 (100)	$[M-H]^-$	**577.1342 (100)**, 559.1199 (19), 533.1453 (21), 439.1028 (25), 425.0892 (11), 393.0971 (11)	C-β_{AB}-EC/EC-β_{AB}-C
	1	5.2	1.067	577.1340 (100)<br>1155.2779 (31)	$[M-H]^-$<br>$[2M-H]^-$	**577.1348 (100)**, 439.1038 (30), 425.0854 (13), 393.0976 (17)	C-β_{AB}-C
			1.142 [a]	577.1345 (100)	$[M-H]^-$	**577.1342 (100)**, 559.1259 (4), 533.1440 (10), 439.1031 (57), 425.0870 (11), 393.0984 (18)	C-β_{AB}-C [a,d]
			1.192	577.1327 (100)<br>667.1301 (38)	$[M-H]^-$<br>$[(M-2H)+2HCOOH-H]^-$	m.d.	
	2	5.3	1.071	577.1350 (100)<br>1155.2766 (7)	$[M-H]^-$<br>$[2M-H]^-$	**577.1342 (100)**, 559.1239 (5), 533.1446 (13), 439.1032 (12), 425.0882 (20), 393.0966 (12)	C-β_{AB}-C [d]
			1.109	577.1348 (100)<br>1155.2764 (20)	$[M-H]^-$<br>$[2M-H]^-$	**577.1354 (100)**, 559.1241 (10), 533.1428 (21), 439.1008 (54), 425.0891 (27), 393.0951 (44)	C-β_{AB}-C [d]
			1.134	577.1349 (100)	$[M-H]^-$	**577.1370 (100)**, 533.1459 (21), 439.1031 (65)	C-β_{AB}-C [d]
			1.150 [a]	577.1344 (100)	$[M-H]^-$	**577.1353 (100)**, 439.1031 (39), 425.0871 (17), 393.0979 (14)	C-β_{AB}-C [a,d]
			1.186	577.1344 (48)<br>599.1143 (7)<br>667.1295 (100)<br>1155.2768 (42)	$[M-H]^-$<br>$[(M-H)-H+Na]^-$<br>$[(M-2H)+2HCOOH-H]^-$<br>$[2M-H]^-$	m.d.	
	3	5.4	1.111	577.1361 (100)	$[M-H]^-$	**577.1327 (100)**, 559.1262 (14), 439.1068 (84), 425.0868 (17)	C-β_{AB}-C [d]
	4	5.5	1.081	577.1337 (100)	$[M-H]^-$	m.d.	
	5	5.8	1.097	577.1349 (100)	$[M-H]^-$	**577.1323 (100)**, 533.1461 (15), 439.1030 (14), 425.0866 (12), 393.0955 (5)	C-β_{AB}-C [d]
			1.128 [a]	577.1341 (100)	$[M-H]^-$	**577.1366 (100)**, 533.1464 (6), 439.0994 (32), 425.0885 (9)	C-β_{AB}-EC/EC-β_{AB}-C [a]
			1.186	577.1338 (100)	$[M-H]^-$	m.d.	C-β_{AB}-C [d]
	26	6.0	1.112	577.1345 (100)	$[M-H]^-$	**577.1335 (100)**, 559.1207 (14), 533.1455 (7), 439.1035 (40), 425.0834 (41), 393.1014 (23)	C-β_{AB}-EC/EC-β_{AB}-C
	16	6.2	1.105	577.1358 (100)	$[M-H]^-$	**577.1298 (100)**, 533.1501 (18), 439.1058 (26), 425.0904 (42)	EC-β_{AB}-EC
			1.161 [a]	577.1338 (100)	$[M-H]^-$	**577.1372 (100)**, 559.1201 (15), 533.1464 (13), 439.1000 (26), 425.0863 (44)	C-β_{AB}-EC/EC-β_{AB}-C [a]

Table S2 : Continued

Sample	Peak No	Rt (min)	Mobility ($1/K_0$)	Ion (m/z) (relative abundance %) [b,c]	Ion identity proposal	MS/MS fragments of $[M{-}H]^-$ (relative abundance %)	Tentative annotation[d, e, f]
(+)-catechin/(−)-epicatechin oxidation model	27	6.4	1.159	577.1350 (100)	$[M{-}H]^-$	**577.1361 (100)**, 559.1281 (21), 439.1019 (28), 425.0888 (42), 393.1003 (7)	C-β_{AB}-EC/EC-β_{AB}-C
	6	6.5	1.124	577.1330 (100)	$[M{-}H]^-$	**577.1340 (100)**, 533.1490 (13), 439.1040 (22), 393.1003 (55)	C-β_{AB}-C/epimer of that compound
	7	6.7	1.060	577.1343 (100)	$[M{-}H]^-$	577.1417 (19), **289.0770 (100)**	C-ε_{AB}-C [d]
			1.078 [a]	577.1348 (100)	$[M{-}H]^-$	**577.1409 (100)**, 439.1021 (21), 425.0853 (21), 289.0702 (85)	C-ε_{AB}-C [a,d]
	28	6.9	1.083	577.1338 (100)	$[M{-}H]^-$	**577.1340 (100)**, 559.1222 (35), 533.1406 (10), 439.1015 (34), 425.0859 (30), 393.0961 (6)	C-β_{AB}-EC/EC-β_{AB}-C
	17	7.2	1.067	577.1349 (100)	$[M{-}H]^-$	**577.1356 (100)**, 533.1472 (11), 439.1027 (39), 425.0874 (18), 393.0987 (15)	EC-β_{AB}-EC
			1.149	577.1344 (100)	$[M{-}H]^-$	**577.1329 (100)**, 533.1391 (16), 425.0933 (12)	EC-β_{AB}-EC
			1.173 [a]	577.1343 (100)<br>667.1303 (5)	$[M{-}H]^-$<br>$[(M{-}2H){+}2HCOOH{-}H]^-$	**577.1370 (100)**, 559.1220 (10), 439.1061 (23), 425.0889 (25)	EC-β_{AB}-EC [a]
			1.209	577.1349 (100)<br>599.1160 (71)<br>667.1293 (14)	$[M{-}H]^-$<br>$[(M{-}H){-}H{+}Na]^-$<br>$[(M{-}2H){+}2HCOOH{-}H]^-$	m.d.	EC-β_{AB}-EC
	29	7.6	1.141	577.1341 (100)	$[M{-}H]^-$	**577.1531 (100)**, 393.0993 (94)	C-β_{AB}-EC/EC-β_{AB}-C
	18	8.1	1.066	577.1341 (100)	$[M{-}H]^-$	**577.1355 (100)**, 559.1242 (10), 533.1442 (10), 439.1002 (32), 425.0870 (28), 393.0991 (21)	EC-β_{AB}-EC
			1.130 [a]	577.1342 (100)	$[M{-}H]^-$	**577.1331 (100)**, 559.1193 (9), 533.1431 (6), 439.1032 (55), 425.0870 (22), 393.0959 (18)	EC-β_{AB}-EC [a]
	8	9.2	1.086	577.1341 (100)	$[M{-}H]^-$	**577.1364 (100)**, 439.1072 (12), 289.0727 (42)	C-β_{AB}-C [d]/C-ε_{AB}-EC/EC-ε_{AB}-C
	30	9.8	1.033	577.1344 (74)	$[M{-}H]^-$	425.0901 (5), **289.0694 (100)**	C-ε_{AB}-EC/EC-ε_{AB}-C
			1.055	577.1326 (98)	$[M{-}H]^-$	577.1297 (81), **289.0715 (100)**	C-ε_{AB}-EC/EC-ε_{AB}-C
			1.075 [a]	577.1343 (100)	$[M{-}H]^-$	577.1303 (43), 289.0690 (56), **287.0560 (100)**	C-ε_{AB}-EC/EC-ε_{AB}-C [a]
	19	10.1	1.052	577.1347 (100)	$[M{-}H]^-$	577.1417 (23), **289.0675 (100)**	EC-ε_{AB}-EC
			1.077 [a]	577.1335 (100)	$[M{-}H]^-$	577.1303 (39), 425.0901 (27), **289.0733 (100)**	EC-ε_{AB}-EC [a]
	9	10.1	1.174	577.1366 (100)	$[M{-}H]^-$	m.d.	C-ε_{AB}-EC/EC-ε_{AB}-C
	10	10.2	1.196	577.1346 (100)	$[M{-}H]^-$	**577.1324 (100)**, 425.0868 (6)	C-β_{AB}-C [d]
	31	10.5	1.080	577.1341 (57)	$[M{-}H]^-$	**577.1313 (100)**, 26 533.1427 (48), 439.1040 (62)	C-β_{AB}-EC/EC-β_{AB}-C
	11	10.9	1.094	577.1344 (100)	$[M{-}H]^-$	577.1334 (14), 533.1464 (4), 439.1070 (8), **289.0725 (100)**	C-ε_{AB}-C [d]
	32	11.1	1.196	577.1342 (100)	$[M{-}H]^-$	**577.1353 (100)**, 439.1038 (16), 425.0868 (18)	C-β_{AB}-EC/EC-β_{AB}-C
	33	11.5	1.200	577.1346 (100)	$[M{-}H]^-$	**577.1319 (100)**, 533.1449 (33), 439.1013 (50), 425.0897 (43)	C-β_{AB}-EC/EC-β_{AB}-C

Table S2 : Continued

Sample	Peak No	Rt (min)	Mobility ($1/K_0$)	Ion (m/z) (relative abundance %) [b,c]	Ion identity proposal	MS/MS fragments of $[M-H]^-$ (relative abundance %)	Tentative annotation [d, e, f]
(+)-catechin/(−)-epicatechin oxidation model	34	11.9	1.094	577.1357 (100)	$[M-H]^-$	577.1359 (15), 439.1005 (4), 425.0887 (4), **289.0721 (100)**	C-ε_{AB}-EC/EC-ε_{AB}-C
	20	12.2	1.097	577.1348 (50)	$[M-H]^-$	m.d.	
			1.197 [a]	577.1340 (100)	$[M-H]^-$	m.d.	
	21	13.1	1.099	577.1343 (100)	$[M-H]^-$	577.1342 (44), 439.1047 (15), 425.0868 (19), **289.0716 (100)**	EC-ε_{AB}-EC
	12	12.2	1.095	575.1190 (100)	$[M-H]^-$	**575.1182 (100)**, 533.1099 (10), 439.1032 (20), 425.0900 (1), 394.0691 (45), 393.0993 (4), 289.0702 (40)	
				1151.2450 (28)	$[2M-H]^-$		
			1.176	575.1185 (100)	$[M-H]^-$	**575.1189 (100)**, 449.0864 (10), 437.0870 (12), 423.0714 (12), 407.0778 (12), 394.0690 (57)	C-γ_{AB}-EC/EC-γ_{AB}-C
	14	13.2	1.098	575.1195 (100)	$[M-H]^-$	**575.1200 (100)**, 449.0878 (14), 437.0899 (17), 407.0744 (32), 394.0689 (51), 289.0735 (39)	
				1151.2453 (18)	$[2M-H]^-$		
			1.129	575.1187 (100)	$[M-H]^-$	**575.1183 (100)**, 449.0856 (14), 423.0694 (11)	C-γ_{AB}-EC/EC-γ_{AB}-C/C-δ_{AB}-EC/EC-δ_{AB}-C
			1.169 [a]	575.1190 (100)	$[M-H]^-$	**575.1188 (100)**, 449.0868 (15), 437.0870 (6), 423.0710 (8), 407.0756 (11), 394.0688 (42)	C-γ_{AB}-C [d]
	35	13.4	1.024	575.1193	$[M-H]^-$	m.d.	
				1151.2452 (62)	$[2M-H]^-$		
			1.122	575.1189	$[M-H]^-$	**575.1200 (100)**, 449.0861 (8), 437.0882 (4), 407.0752 (3), 394.0684 (10)	C-γ_{AB}-EC/EC-γ_{AB}-C
				1151.2452 (10)	$[2M-H]^-$		
			1.156 [a]	575.1194 (100)	$[M-H]^-$	**575.1193 (100)**, 449.0878 (12), 437.0872 (9), 423.0723 (8), 407.0757 (9), 394.0689 (17)	C-γ_{AB}-EC/EC-γ_{AB}-C [a]
	22	13.6	1.208	575.1192 (100)	$[M-H]^-$	**575.1178 (100)**, 449.0882 (24), 437.0899 (5), 423.0733 (19), 407.0746 (16), 394.0694 (5)	EC-γ_{AB}-EC
	23	14	1.123	575.1194 (100)	$[M-H]^-$	**575.1231 (100)**, 449.0835 (36)	EC-γ_{AB}-EC/EC-δ_{AB}-EC
	24	14.2	1.124	575.1193 (100)	$[M-H]^-$	**575.1185 (100)**, 449.0864 (11)	
			1.168 [a]	575.1190 (100)	$[M-H]^-$	**575.1194 (100)**, 449.0868 (13), 437.0882 (6), 423.0683 (6), 407.0734 (9), 394.0689 (17)	EC-γ_{AB}-EC [a]
Grape seed extract	25	4.7	1.108	577.1348 (100)	$[M-H]^-$	**577.1354 (100)**, 559.1222 (40), 533.1444 (33), 439.1013 (13), 425.0860 (46), 393.0976 (18)	C-β_{AB}-EC/EC-β_{AB}-C
	1	5.2	1.139	577.1342 (100)	$[M-H]^-$	**577.1329 (100)**, 559.1257 (5), 533.1461 (5), 439.1024 (45), 425.0870 (13), 393.0988 (24)	C-β_{AB}-C [d]
	2	5.3	1.075	577.1342 (100)	$[M-H]^-$	577.1364 (8), 451.1022 (22), 425.0872 (30), **407.0766 (100)**, 289.0714 (94)	Procyanidin B3 [d]
			1.093 [a]	577.1341 (100)	$[M-H]^-$	577.1336 (13), 451.1026 (22), 425.0867 (34), 407.0766 (97), **289.0714 (100)**	Procyanidin B1 [a, d]
			1.137	577.1339 (100)	$[M-H]^-$	577.1339 (78), 533.1458 (34), **407.0770 (100)**, 289.0715 (43)	Procyanidin B1 protomer [d]
				667.1300 (28)	$[(M-2H)+2HCOOH-H]^-$		

Table S2 : Continued

Sample	Peak No	Rt (min)	Mobility (1/K$_0$)	Ion (*m/z*) (relative abundance %) [b,c]	Ion identity proposal	MS/MS fragments of [M−H]$^-$ (relative abundance %)	Tentative annotation[d, e, f]
Grape seed extract	26	5.9	1.114	577.1343 (53)	[M−H]$^-$	**577.1316 (100)**, 533.1458 (4), 451.1065 (18), 439.1022 (33), 425.0861 (28), 393.0942 (15)	C-β_{AB}-EC/EC-β_{AB}-C/PC type
	16	6.2	1.077	577.1340 (100)	[M−H]$^-$	577.1331 (14), 451.1027 (18), 425.0869 (37), **407.0764 (100)**, 289.0713 (92)	Procyanidin B4 [d]
	6	6.5	1.120	577.1340 (100)	[M−H]$^-$	577.1336 (51), 559.1256 (8), 451.1026 (14), 439.1044 (15), 425.0863 (23), 407.0769 (21), 393.0976 (7), **289.0698 (100)**	C-β_{AB}-C/epimer of that compound /PC type
	7	6.6	1.093	577.1338 (100)	[M−H]$^-$	577.1345 (14), 451.1025 (20), 425.0867 (40), 407.0762 (94), **289.0714 (100)**	Procyanidin B2 [d]
	17	7.1	1.148 [a]	577.1336 (100)	[M−H]$^-$	**577.1342 (100)**, 425.0896 (34)	EC-β_{AB}-EC [a]
			1.176	577.1344 (100)<br>667.1286 (31)<br>599.1166 (9)	[M−H]$^-$<br>[(M−2H)+2HCOOH−H]$^-$<br>[(M−H)−H+Na]−	**577.1289 (100)**, 425.0882 (45)	EC-β_{AB}-EC
	29	7.5	1.142	577.1329 (100)	[M−H]$^-$	**559.1218 (100)**, 439.1034 (55), 393.0958 (81)	C-β_{AB}-EC/EC-β_{AB}-C
	18	8.1	1.131	577.1337 (100)	[M−H]$^-$	**577.1347 (100)**, 559.1218 (4), 439.1028 (45), 393.0975 (27)	EC-β_{AB}-EC
	8	9.1	1.087	577.1334 (57)	[M−H]$^-$	**577.1307 (100)**, 559.1284 (24), 451.1030 (13), 439.1003 (79), 425.0889 (36), 407.0742 (12), 289.0671 (85)	C-β_{AB}-C [d]/PC type
	31	10.5	1.080	577.1338 (100)	[M−H]$^-$	**577.1334 (100)**, 533.1440 (33), 451.1064 (5), 439.1024 (63), 425.0864 (10), 407.0722 (29), 289.0708 (20)	C-β_{AB}-EC/EC-β_{AB}-C/PC type
	34	11.8	1.144	577.1348 (100)	[M−H]$^-$	577.1306 (20), 451.1036 (15), 425.0877 (33), 407.0776 (40), **289.0719 (100)**	Procyanidin B5 [d]
	21	13.1	1.098	577.1335 (100)	[M−H]$^-$	**577.1360 (100)**, 289.0724 (78)	EC-ε_{AB}-EC

[a] Correspond to compounds showing the most abundant MS and MS/MS signals among the other compounds of a same chromatographic peak. [b] Only adduct ions showing relative intensity >5% are shown. [c] *m/z* 577 and *m/z* 575 corresponded to [C30H25O12]$^-$ and [C30H23O12]$^-$, respectively ; *m/z* 1155 and *m/z* 1151 corresponded to [C60H51O24]$^-$ and [C60H47O24]$^-$, respectively ; *m/z* 667, *m/z* 645 and *m/z* 599 corresponded to [C32H27O16]$^-$, [C31H26NaO14]$^-$ and [C30H24NaO12]$^-$, respectively (ppm error <5.6). [d] Confirmed with reference compounds (see Figure 1 and Table S1 for structures and spectral data). [e] Dehydrodicatechin nomenclature is described in Verloop and co-workers [1]. [f] When annotation is ambiguous, the different options are separated by "/". **m.d.** : missing data. **PC** : procyanidin. **C** : (+)-catechin. **EC** : (−)-epicatechin

References

1. Verloop, A.J.W.; Gruppen, H.; Vincken, J.-P. Annotation of Different Dehydrocatechin Oligomers by MS/MS and Their Occurrence in Black Tea. *J. Agric. Food Chem.* **2016**, *64*, 6011–6023, doi:10.1021/acs.jafc.6b01695.
